# Implementing at-scale, community-based distribution of misoprostol tablets to mothers in the third stage of labor for the prevention of postpartum haemorrhage in Sokoto State, Nigeria: Early results and lessons learned

**DOI:** 10.1371/journal.pone.0170739

**Published:** 2017-02-24

**Authors:** Nosakhare Orobaton, Jumare Abdulazeez, Dele Abegunde, Kamil Shoretire, Abubakar Maishanu, Nnenna Ikoro, Bolaji Fapohunda, Wapada Balami, Katherine Beal, Akeem Ganiyu, Ringpon Gwamzhi, Anne Austin

**Affiliations:** 1 JSI Research &Training Institute, Inc., Boston, Massachusetts, United States of America; 2 USAID/Targeted States High Impact Project (TSHIP), Sokoto, Nigeria; 3 Department of Family Health, Federal Ministry of Health, Abuja, Nigeria; Indiana University School of Medicine, UNITED STATES

## Abstract

**Background:**

Postpartum haemorrhage (PPH) is a leading cause of maternal death in Sokoto State, Nigeria, where 95% of women give birth outside of a health facility. Although pilot schemes have demonstrated the value of community-based distribution of misoprostol for the prevention of PPH, none have provided practical insight on taking such programs to scale.

**Methods:**

A community-based system for the distribution of misoprostol tablets (in 600ug) and chlorhexidine digluconate gel 7.1% to mother-newborn dyads was introduced by state government officials and community leaders throughout Sokoto State in April 2013, with the potential to reach an estimated 190,467 annual births. A simple outcome form that collected distribution and consumption data was used to assess the percentage of mothers that received misoprostol at labor through December 2014. Mothers’ conditions were tracked through 6 weeks postpartum. Verbal autopsies were conducted on associated maternal deaths.

**Results:**

Misoprostol distribution was successfully introduced and reached mothers in labor in all 244 wards in Sokoto State. Community data collection systems were successfully operational in all 244 wards with reliable capacity to record maternal deaths. 70,982 women or 22% of expected births received misoprostol from April 2013 to December 2014. Between April and December 2013, 33 women (< 1%) reported that heavy bleeding persisted after misoprostol use and were promptly referred. There were a total of 11 deaths in the 2013 cohort which were confirmed as maternal deaths by verbal autopsies. Between January and December of 2014, a total 434 women (1.25%) that ingested misoprostol reported associated side effects.

**Conclusion:**

It is feasible and safe to utilize government guidelines on results-based primary health care to successfully introduce community distribution of life saving misoprostol at scale to reduce PPH and improve maternal outcomes. Lessons from Sokoto State’s at-scale program implementation, to assure every mother’s right to uterotonics, can inform scale-up elsewhere in Nigeria.

## Introduction

Nigeria’s Maternal Mortality Ratio (MMR) declined from an estimated 1,350 maternal deaths per 100,000 live births in 1990 to an estimated 814 maternal deaths per 100,000 live births in 2015.[1] Recent data indicate that national MMR estimates remained largely unchanged between 2010 and 2015. There were an estimated 58,000 maternal deaths in 2015. Nigeria had 2.5% of the world’s population, yet accounted for 19% of the global burden of maternal mortality.[1]

In 2013, 64% of women in Nigeria delivered their most recent babies at home. In such a setting, mothers do not typically have access to lifesaving uterotonics. A sub-population of these women who deliver at home, do so alone, with no one present (NOP).[2,3] In 2013, women who delivered with NOP accounted for an estimated one million births in Nigeria.[4]^,^[5] Women that cannot, or do not, gain timely access to facility-based skilled care have a greater risk of dying from PPH or other life-threatening causes of avoidable maternal mortality.

Between 2003 and 2009, PPH was globally estimated to account for over 19% of maternal deaths. In Sub-Saharan Africa, an estimated 15.2% of all maternal deaths were directly attributable to PPH.[6] The majority of PPH events and deaths could have been averted through the “prophylactic use of uterotonics during the third stage of labor.”[7] Oxytocin is the preferred uterotonic. When oxytocin is unavailable, the World Health Organisation (WHO) recommends the use of “other injectable uterotonics … or oral misoprostol (600 μg).”[7] Recent studies have confirmed that misoprostol is not inferior in performance to oxytocin as a uterotonic.[8] In 2006, Nigeria was the first country to approve the use of misoprostol for PPH prevention in community settings. Misoprostol was included in the 5^th^ revision of the national essential medicines list in 2010.[9]^,^[10] The WHO confirmed the inclusion of misoprostol with the 18^th^ revision of the WHO List of Essential Medicines.[11]^,^[12]

Misoprostol, which is heat stable, of low cost and easy to administer orally, has been proposed as the best way to administer a uterotonic in deliveries that occur outside of health facility settings.[13] One such route, community-based distribution, has shown that misoprostol can be safely and appropriately administered by community health workers.[14,15] Community-based distribution has also been associated with decreased prevalence of PPH among misoprostol users.[15–20] Based on evidence from pilot studies on community-based distribution, the WHO now recommends that trained lay health workers may be utilized to administer misoprostol to prevent PPH. [21]

While useful as platforms to establish proof of concept, pilot studies are limited in informing “how” to take programs to scale in real-world settings. Specifically, operational issues uniquely associated with at-scale implementation may not be uncovered nor are they sufficiently well understood in pilot settings. We posit that a better understanding of community distribution efforts at scale is best informed by insights and experiences obtainable from operations in real-life settings, conducted at scale.

This paper is a companion to an earlier publication on the roll-out and scale up of chlorhexidine digluconate 7.1% gel for the prevention of newborn cord infection in Sokoto State.[22] Although misoprostol and chlorhexidine were distributed together, in one program, to mothers and their newborns as a dyadic unit, a separate treatment of misoprostol is warranted for a number of reasons. First, unlike chlorhexidine, its adoption imposed unique program design considerations prompted by Islamic leaders’ reconciliation of newly learned information about the benefits of misoprostol in the prevention of PPH with fears that the medicine could also be used as an abortifacient early in pregnancy. Second, unlike the chlorhexidine paper, we focused on a two-year period to test for early signals of changes in community demand which could serve as a more robust measure of community acceptance. Third, although misoprostol was nationally approved for community-based distribution in Nigeria, its use is still confined to health facilities. We posit that a paper that specifically discusses experiences with its use at scale, could likely contribute much needed insight for scale-up efforts elsewhere in Nigeria, Sub-Saharan Africa and globally.

### Objectives of paper

Between 2010 and 2015, the United States Agency for International Development (USAID)-funded Targeted States High Impact Project (TSHIP), managed by JSI Research & Training Institute, Inc. (JSI), provided technical support to the Sokoto State Government to initiate and expand community-based delivery of misoprostol statewide, at scale. We highlight the critical programmatic steps undertaken to secure community trust and ownership. We also share tools developed and the approaches used to monitor misoprostol distribution and related maternal outcomes. Early lessons learned and results are presented.

### The program setting

Sokoto State is situated in the northwest corner of Nigeria, bordered by Birnin Kebbi State to the southeast and Zamfara State to the south. It shares international borders with the Benin Republic to the west and Niger Republic to the north.[23] The population of the state in 2013 was estimated to be just over 4.6 million.[24] In 2009, the MMR in Sokoto State was estimated to be 1,500 deaths per 100,000 live births.[24] In 2008 and 2013, 95% of women in Sokoto State reported having delivered their most recent child at home.[25,26] In 2013, 84% of women reported that they were assisted by a traditional birth attendant; 8% were assisted by family members. [26] In 2013, more than 80% of women still reported non-use of antenatal care in a most recent pregnancy.[26]^, 3^ Although there are no direct estimates of the burden of PPH in Sokoto State, a recent study in Bauchi State in Northeastern Nigeria—similar in socio-economic profile to Sokoto State—found that 23% of women in primary level care centers and 41% of women at secondary level care and higher facilities, suffered from PPH. In the study, PPH accounted for 19% of deaths due to direct complications at primary level care facilities, and to 9% of deaths in secondary level care or higher facilities.[27] The use of misoprostol in the Sokoto State context has been limited to health facility settings, as a substitute for oxytocin. Heretofore, its use in home-based deliveries was precluded by the absence of a system for community-based distribution at the household level.

### Developing the at-scale, community-based, misoprostol distribution system

In May 2012, TSHIP consulted with Sokoto State Ministry of Health (SMOH) and the Ministry of Local Government (MOLG) and Chieftaincy Affairs, to introduce and facilitate an at-scale community distribution of misoprostol. At the time, the MOLG was responsible for the management of health personnel and primary health centers and dispensaries. The MOLG also supervised Local Government Council leaders, and had direct access to district and ward heads that managed ward development committees (WDC). The SMOH oversaw the delivery of services in secondary level facilities. The SMOH also played a role in technical training of health workers based in primary level centers. This fragmentation of the health sector by the division of labor between the MOLG and the MOH, was a legacy of Sokoto State government’s initial lack of progress in the implementation the primary health care-under-one-roof (PHCUOR) policy. The PHCUOR policy of the National Primary Health Care Development Agency (NPHCDA), was designed to unite all primary health care under the State Primary Health Care Development Agency (SPHCDA). Sokoto State adopted PHCUOR through an act of legislation in early 2016.

In October 2012, the Sokoto State government funded and procured 56,832 doses of misoprostol from the Nigeria office of Marie Stopes International.[28] This was the first recorded instance of government financing of misoprostol use for community distribution in Nigeria. In 2014, the Sokoto State government procured an additional 56,000 doses of misoprostol.[29]

### Gaining community trust

In an environment still beset by mistrust associated with polio vaccination campaigns, several steps were carefully taken to gain community trust and pre-emptively quell potential misinformation about misoprostol.[30,31] The MOLG consulted with all 244 WDC chairmen who were briefed on PPH and neonatal mortality and newborn cord infections. The chairmen were briefed on the program steps and the roles of each set of actors including WDC chairmen, Local Government Area (LGA) and state government ministries. The MOLG also worked with the influential and trusted Nigeria Aid Group (NAG), the charity arm of Jama’atu Nasril Islam (JNI), Nigeria’s largest Islamic non-profit organization. Leaders and members of NAG/JNI include district heads, hospital-based physicians and nurses, Islamic clerics, and community health extension workers. Its leadership was already aware of the importance of misoprostol to prevent PPH. NAG/JNI leaders conveyed their support for the program to all 23 LGA Council chairmen in the state, with district heads, imams and clerics in mosques and at wedding celebrations and baby naming ceremonies. Men were specifically targeted in these advocacy efforts as husbands’ approval—in the Sokoto State context—is required prior to wives’ acceptance to use medicines.

**Fig 1:**
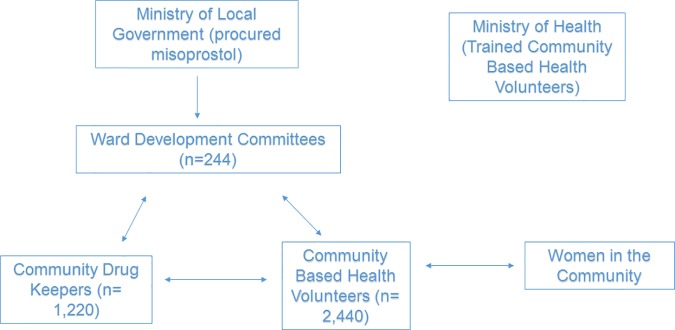
Pathway to community based distribution of misoprostol in Sokoto State.

Whilst there was strong support to use misoprostol to prevent PPH, community leaders were already aware it could be used in much larger quantities, to terminate pregnancy. To preemptively ensure that misoprostol was used only for PPH prevention, and to gain the support of male heads of households, community leaders insisted in the creation of a volunteer community drug keeper (CDK). The introduction of CDK, a “satisfice” decision, made misoprostol acceptable to traditional leaders for community-based distribution. The CDK, almost always a man, was chosen by the WDC on account of being trustworthy, reliable, and available twenty-four hours a day, to release medicines on demand. CDK were also required to be able read, write and keep simple records. The agreed-upon criterion to dispense misoprostol was verbal notification by a family member or the community-based health volunteer (CBHV) that a woman was in labor. Notwithstanding the risks of likely additional delays in delivering misoprostol by the added CDK layer, it was determined that it was better to have a distribution on demand program, with its flaws, than none at all. Each of the 244 wards in Sokoto had 5 CDKs. Among the 1220 CDK’s selected throughout the 244 wards, 33 (2.7%) were female. Fig 1 presents the flow of commodities from the state level to the community level.

Each WDC chairman proactively watched for early signs of community opposition or misinformation, and regularly held facts-based discussions with affected male heads of households.

### Investing in the community distribution system

A cadre of CBHVs were identified and trained. The CBHV concept was guided by the Ward Minimum Package of Services guidelines of the NPHCDA.[32] Selection criteria were formulated by community leaders to appoint CBHVs. These included being female, being respected by the community, being resident in the ward where they would work, and having a record of dependability. In 2012, 2,440 all-female CBHVs were selected and trained on counseling, the delivery of 18 key health messages (Table 1), and simple technologies such as oral rehydration therapy. They were also trained to counsel and demonstrate the proper use (dosage and timing of administration) of misoprostol; this was a part of the maternal health package. CBHVs also actively promoted facility-based delivery.

**Table 1:** CBHV messages to pregnant women transmitted through pictorial counselling cards.

**1**	Importance of antenatal clinic attendance for pregnant women
**2**	Importance of screening tests, including HIV in pregnancy
**3**	Importance of intermittent preventive therapy for malaria in pregnancy using Sulphadoxine-pyrimethamine tablets
**4**	Importance of skilled attendance in labor and postpartum care
**5**	Importance of an insecticide-treated net and how to use it at home
**6**	Types of food, importance of an adequate and balanced diet in pregnancy, and vitamin A supplementation for children
**7**	The importance of iron and folic acid during pregnancy
**8**	Importance of using misoprostol tablets after delivery of the baby and how to use it
**9**	The importance of chlorhexidine gel for cord care and how to apply it on the cord; Avoiding use of potentially harmful substances to the cord
**10**	The care for low birth weight babies with Kangaroo care
**11**	How to prepare the Oral Rehydration Solution (ORS) for diarrheal diseases in children under 5 years, and the importance of ORS.
**12**	Importance of immunization and when to get them, including the tetanus toxoid for mothers
**13**	Importance of birth preparedness and emergency plan, including transport for referrals
**14**	Danger signs in pregnancy, and identification and prompt referral to a health facility if they occur
**15**	Danger signs in the newborns, and identification and prompt referral to a health facility if they occur
**16**	The use of artemisinin-based combination antimalarial drugs and how to use the tablets for children
**17**	Importance of exclusive breastfeeding and how to put baby to breast for optimal growth and weight gain by the child
**18**	Importance of hand-washing and general hygiene in the home

With commencement of labor, families notified the CBHV who in turn obtained a package of misoprostol from the CDK that was subsequently delivered to the mother. The CBHV was expected to directly observe the mother ingesting misoprostol tablets in the third stage of labor, after the delivery of the baby and prior to the delivery of the placenta. State and LGA authorities strengthened the capabilities of the WDC to manage health and development activities within their jurisdiction. The NPHCDA Ward minimum package specified a central role for WDCs in the governance of health and social sector interventions at the ward level.[32] WDCs were trained on rural participatory methodologies to foster community ownership, organizational development, leadership and recordkeeping.

## Methods

A simple patient outcome form was designed and used by CDKs and CBHVs, with assistance from facility-based health workers, to collect a limited set of data. This form collected the patient’s name, the name of the patient’s village, date of delivery, whether or not misoprostol was ingested, and the condition of the mother at birth during the intrapartum period. At 42 days postpartum, data were collected on whether or not the mother was alive. In January 2014 the project also began to collect data on complications, in the intrapartum period, associated with misoprostol ingestion. Data collection was limited to the intrapartum and 42 day postpartum period.

Data were collected at the community level through observation and questioning by the CBHV and transmitted verbally to the CDK, who entered the information into the outcome forms. Monthly review meetings were held with CDKs, CBHVs and health facility leadership staff in each of the wards and inconsistencies in the data were regularly identified and addressed. Collated data from the patient outcome form were collected by TSHIP and each record was double-entered using Epi-Data software. Data were then transferred into STATA® software (version 11.0) for analyses. Both the 2013 and 2014 data were verified by the National Population Commission (NPC) Office of Sokoto State through home visits and a two-step process. First, the status of women listed as dead was double-checked. Next, a 5% random sample of all mothers that had received misoprostol were visited in their homes. There was an observed error rate of 1.6%. Corrections were made to all the affected records in the master database before the analysis.

Within the 2013 cohort of mothers, each woman that died during the 42-day postpartum period was located by name, ward, address and LGA. Maternal deaths were only verified for women who had resided in the intervention LGAs. The WHO-based forms were used to conduct verbal autopsies on each woman confirmed as dead. The first step was to make inquiries about women of reproductive age who may have died of maternal-related causes and may not have been included in the count of deaths. No women were found to be omitted.

After identifying the women, the verbal autopsy process continued with separate meetings with local government and community officials. Thereafter, the CDK was contacted. The CDK identified the residence of the deceased and secured reliable informants. If female participants wanted to be interviewed in their homes, the female researcher would do so. Three medical doctors trained in cause-of-death certification, using International Classification of Disease, Tenth Edition (ICD10) coding, worked independently to determine the causes of deaths.

Ethical clearance for the study was granted by Sokoto Government Health Research Ethics Committee, Nigeria. Informed consent was obtained from all respondents after the purpose of the study was explained and after they understood that they were free to choose not to participate, or withdraw at any time from the study without any risk. JSI’s Institutional Review Board (IRB) also granted approval to use the anonymized program data collected in the outcome forms for this analysis.

## Results

The 244 WDCs successfully set up by the MOLG commenced a community distribution system that delivered misoprostol through the CBHV/CDK to women who delivered at home throughout Sokoto State. Similarly, community data collection systems that reliably collected maternal death data were successfully installed in all 244 wards. In addition, monthly meetings at the ward level were regularly held in all 244 wards to appraise program performance. Data from two learning periods, April to December 2013 and January to December 2014, were analyzed. Based on official population projections, there were an estimated expected 138,196 live births between April and December 2013. A total of 36,370 mothers or 26.3% of all births received misoprostol via community distribution. The highest period coverage was found in Kware LGA at 50%, the lowest in Isa LGA at 17%. There was an inverse association between LGA population size and their respective percent misoprostol coverage; higher population LGAs had a lower proportion of women receiving misoprostol (Table 2) This negative correlation coefficient (-0.70) is mainly a result of an initial decision by the MOLG, as a demonstration of equal consideration, to distribute equal amounts of misoprostol to all wards in all LGAs by the MOLG, irrespective of the population size of women in need. On the basis of this finding, the MOLG revised its plans and adopted a more equitable distribution strategy in 2014 that was based on population size.

**Table 2:** Percentage of Births that received Misoprostol in each Local Government Area (LGA) between April and December of 2013.

**LGA**	**Total number of mothers reached with Misoprostol**	**Expected Births (April-December 2013)**	**Percentage of births covered**
Binji	1,338	4,304	31.1%
Bodinga	1,223	6,432	19.0%
Dange Shuni	1,616	7,030	23.0%
Gada	1,723	9,637	17.9%
Goronyo	1,760	7,095	24.8%
Gudu	1,988	4,264	46.6%
Gwadabawa	1,874	8,792	21.3%
Illela	1,511	4,798	31.5%
Isa	1,018	5,994	17.0%
Kebbe	1,092	5,305	20.6%
Kware	2,137	4,315	49.5%
Rabah	2,259	5,692	39.7%
Sabon Birni	2,375	7,223	32.9%
Shagari	1,482	6,169	24.0%
Silame	1,684	3,956	42.6%
Sokoto North	1,568	8,361	18.8%
Sokoto South	1,658	7,418	22.4%
Tambuwal	1,537	8,361	18.4%
Tangaza	1,648	3,965	41.6%
Tureta	947	2,671	35.5%
Wamakko	1,237	6,065	20.4%
Wurno	1,452	6,145	23.6%
Yabo	1,243	4,204	29.6%
Total	36,370	138,196	26.3%

Correlation between expected number of births and percentage of births covered: -0.70.

There were 190,467 expected live births between January and December 2014. Of these, 34,612 mothers received misoprostol through community-based distribution, equivalent to 18.2% of all births in Sokoto State in 2014 (Table 3). The highest rates of coverage were found in Kware and Tureta LGAs, each with coverage rates of over 30% of mothers reached (Table 3). Although attempts were made to distribute misoprostol more equitably in 2014, a negative correlation between the number of eligible women in each LGA and the percentage of misoprostol consumption persisted (correlation coefficient: -0.67).

**Table 3:** Percentage of Births that received misoprostol in each Local Government Area (LGA) in 2014.

**LGA**	**Total number of mothers reached with Misoprostol**	**Expected Births (Jan.-December 2014)**	**Percentage of births covered**
Binji	1,280	6,001	21.3%
Bodinga	853	8,830	9.7%
Dange Shuni	2,224	9,630	23.1%
Gada	2,147	13,316	16.1%
Goronyo	2,040	9,812	20.8%
Gudu	1,401	6,009	23.3%
Gwadabawa	1,206	12,115	10.0%
Illela	1,547	6,454	24.0%
Isa	1,600	8,317	19.2%
Kebbe	860	7,439	11.6%
Kware	1,774	5,811	30.5%
Rabah	2,079	7,851	26.5%
Sabon Birni	2,458	9,835	25.0%
Shagari	1,355	8,543	15.9%
Silame	1,383	5,448	25.4%
Sokoto North	1,215	11,828	10.3%
Sokoto South	1,213	10,207	11.9%
Tambuwal	1,079	11,482	9.4%
Tangaza	1,208	5,394	22.4%
Tureta	1,125	3,694	30.5%
Wamakko	1,346	8,225	16.4%
Wurno	2,075	8,466	24.5%
Yabo	1,144	5,760	19.9%
**Total**	34,612	190,467	18.2%

Correlation between expected number of births and percentage of births covered: -0.67.

**Fig 2:**
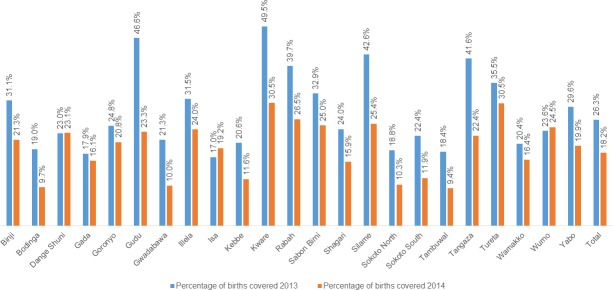
Percentage of mother’s receiving misoprostol, by LGA in two time periods: April-December 2013 and January-December 2014.

**Fig 3:**
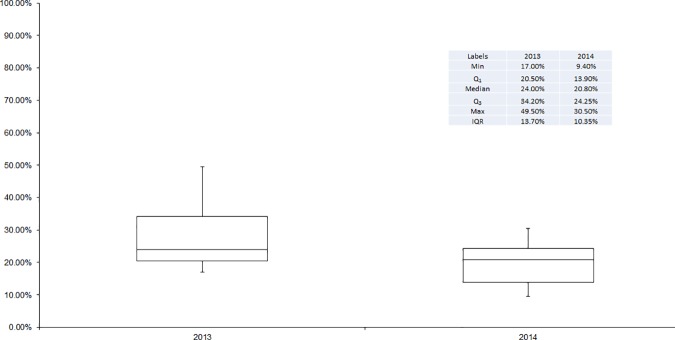
Box plots of the percentage of eligible women receiving Misoprostol in each LGA by year.

Compared to 2013, misoprostol coverage rates dropped in all LGAs in 2014 with the exception of Dange Shuni, Isa and Wormo (Fig 2). The decline ranged from less than 2 percentage points in Gada LGA, to 23 points in Gudu LGA. In 7 LGAs, the drop in coverage exceeded 10 percentage points; in 1 LGA, Gudu, the drop exceeded 20 percentage points. Between these two years, the differentials in LGA level coverage were smaller in 2014 relative to 2013. In 2013, the highest coverage was found in Kware LGA (49.5%) and the lowest was found in Isa LGA (17%), a difference of 32.5 percentage points. In 2014, this difference had declined to 21.5%, with Kware LGA (30.9%) having the highest coverage, and Tambuwal LGA (9.4%) having the lowest coverage. In 2013, there was greater variation in the coverage of women, relative to 2014 (Fig 3). In 2013, differences in coverage between LGAs ranged from 17% to almost 50%. In 2014, this range was much smaller, ranging from 9.4% to 30.5%. Correspondingly, the inter-quartile range (IQR) declined from 13.7% in 2013 to 10.4% in 2014. This indicates that misoprostol was more equitably distributed, by LGA, in 2014 relative to 2013. Median coverage declined from 24% of eligible women in 2013 to 21% of eligible women in 2014.

**Fig 4:**
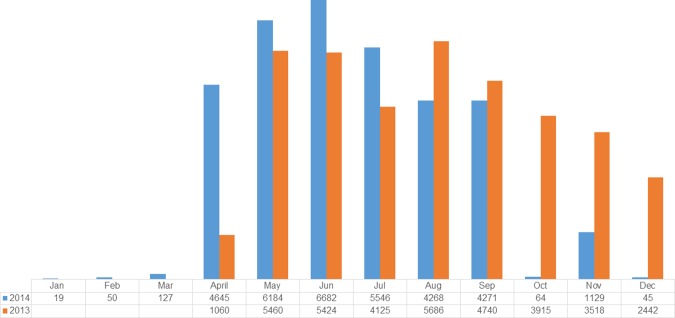
Number of mothers reached with misoprostol in Sokoto State between April 2013 and December 2013 and in 2014 in Sokoto State, Nigeria, by month.

**Fig 5:**
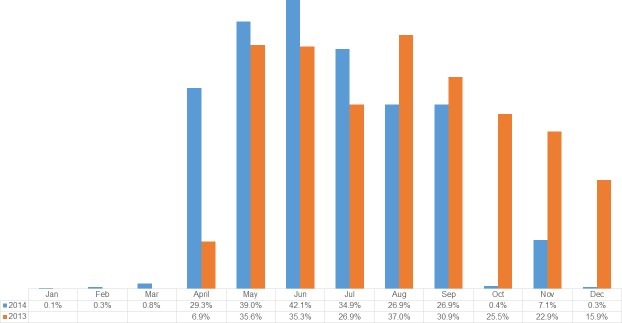
Percentage of mothers reached with misoprostol, based on monthly expected number of births, in Sokoto State between April 2013 and December 2013 and in 2014 in Sokoto State, Nigeria, by month.

**Fig 6:**
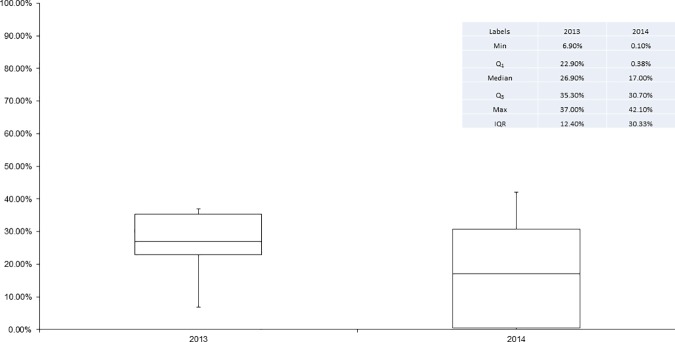
Box plots of the percentage of eligible women receiving Misoprostol by month of distribution and year.

In 2013, the number of mothers that received misoprostol in 2013 peaked in August after a sharp rise in May (Fig 4). There were 3,536 fewer women reached in 12 months in 2014 compared to the 9 month distribution in 2013. In the first three months of 2014, only 197 out of an expected 49,250 mothers received misoprostol. The highest rates of coverage, throughout the project period were in May, June, July and show that between 38% and 42% of mothers, throughout Sokoto State received misoprostol (Fig 5). There was a fourfold increase in the number of women that received misoprostol in April 2014 compared to the same month in 2013. The percentage of eligible women who received misoprostol peaked in May of 2013 (35.6%) and June of 2014 (42.1%) (Fig 6). The lowest levels of coverage, in 2013, were found in April, when the rollout of misoprostol commenced; in 2014 the lowest coverage was found in January, when there were difficulties in procuring the drugs. The IQR in 2013 was 12.4%; in 2014 it increased to over 30%. Median coverage declined from 27% in 2013 to 17% in 2014. Coverage of misoprostol by month, corresponded directly to availability of misoprostol. Higher coverage months indicate better supply of the commodity; lower coverage months indicate months in which misoprostol stock-outs occurred.

**Fig 7:**
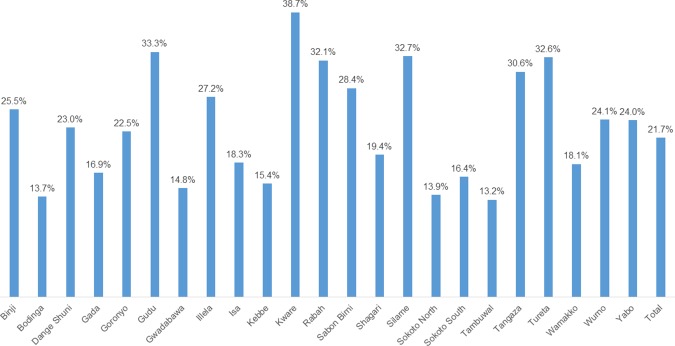
Percentage of mothers that received misoprostol in each Local Government Area (LGA) between April 2013 and December of 2014.

In all, just over 22%, slightly more than one in five mothers, were reached between April 2013 and December 2014 (Fig 7).

### Tracking maternal morbidities and maternal mortality in the 2013 cohort

Among the women served with misoprostol in the 2013 cohort, 33 reported heavy bleeding and were all transferred to health facilities with community-managed emergency transportation schemes. In the same cohort, there were a total of 11 pre-verified maternal deaths all of which were confirmed as maternal-related deaths by verbal autopsies. Table 4 presents the causes and timing of maternal deaths. Eight of the 11 deaths occurred during labor and delivery and the remainder occurred in the postnatal period. Ten of all the deaths occurred at home; the eleventh occurred enroute to the hospital—due to obstructed labor. Three women died prior to the arrival of the CBHV, did not ingest misoprostol, and died of obstructed labor, antepartum hemorrhage and primary PPH. Of the eight women that died and had ingested misoprostol, four died of obstetric hemorrhage, and the remaining died of ruptured uterus, sepsis, eclampsia and acute respiratory infection. Maternal death data among the 2014 cohort was neither collected nor verified during the life of the project.

**Table 4:** Cause of Death as Determined by Verbal Autopsy, including age, parity timing of death and place of delivery.

	**Age**	**Parity**	**Timing of Death**	**Misoprostol ingested**	**Cause of Death**	**Place of delivery**
MAT001	30	6	During labor and delivery	Yes	Pregnancy-related death: Intraperitonic haemorrhage Final diagnosis: Ruptured Uterus—Silent	At home
MAT002	40	5	During labor and delivery	Yes	Pregnancy-related death: Pregnancy Related Sepsis. Likely from premature rupture of membranes	At home
MAT003	45	7	Within 42days after delivery	Yes	Obstetric haemorrhage	At home
MAT004	30	4	During labor and delivery	Yes	Pregnancy-related death: Eclampsia + complications	At home
MAT005	27	5	Within 42days after delivery	Yes	Obstetric haemorrhage	At home
MAT006	27	1	During labor and delivery	No	Pregnancy-related death: Primary Postpartum haemorrhage (PPPH)	At home
MAT007	17	0	During labor and delivery	No	Pregnancy-related death: Antepartum haemorrhage	At home
MAT008	25	4	During labor and delivery	Yes	Obstetric haemorrhage	At home
MAT009	40	7	During labor and delivery	No	Obstructed labor	On the way to hospital
MAT0010	35	1	During labor and delivery	Yes	Other and unspecified maternal CoD: Acute respiratory infection including pneumonia	At home
MAT0011	20	1	Within 42days after delivery	Yes	Obstetric haemorrhage	At home

### Reported misoprostol-related side effects among the 2014 cohort

Among the 34,612 mothers who received misoprostol between January and December of 2014, 98.75% reported no side effects. Of the 434 women reporting side effects, 310 reported only one side effect; 124 women reported two or more side effects. The most common side effect reported was bleeding (n = 185) followed by shivering (n = 177), nausea (n = 157), abdominal cramping (n = 113) and diarrhea (n = 103). These findings are consistent with other studies that have found effects associated with misoprostol are both transient, non-life-threatening and self-resolving.[17]

## Discussion

The decision by Sokoto State government to finance two consecutive procurements of misoprostol is indicative of its willingness to address the high demand for misoprostol, necessary for the prevention of PPH. It also underscores the crucial role of state governments in the delivery of this low-cost, high-impact medicine. In order to ensure adequate supplies of misoprostol for community-based distribution, more needs to be done so that quantification and pipeline analysis based on demographic data are routinely applied. Although a system for reaching mothers “through the last mile” was set up, at least 70% of mothers that delivered at home did not get misoprostol. Three main factors were likely responsible. The first was the substantial under-supply of misoprostol. Formal forecasting exercises, including pipeline analysis, had recommended a higher number of doses than were procured by the government in both 2013 and 2014.

The second was the inequitable distribution of CBHVs linked with widely dispersed populations in some LGAs that may have missed some mothers.[33] The third, also confirmed by a recent study of the program, were delays in the timely release of misoprostol from health facilities to CDK on one hand, and between CDK and women in need, on the other. There were instances of husbands’ rejection of the medicines.[33] However, the majority of men—community leaders and husbands—strongly endorsed misoprostol distribution across all 244 wards in the state. This strong endorsement was further supported by the visible impact of misoprostol on reducing maternal bleeding, which quite likely, further strengthened community demand for misoprostol. In all, the main constraint in delivering misoprostol to women was likely the inadequate supplies in the pipeline.

The strong community demand for misoprostol was reflected by faster consumption of available medicines in 2014 compared to 2013. This strong demand and acceptance of misoprostol has also been confirmed by another study, and communities highlighted its observed quick-acting effect to stop postpartum bleeding.[33] The successful transportation to health centers of 33 women that suffered heavy bleeding, also likely averted PPH-related deaths. Yet, 11 maternal deaths could also potentially have been averted were they referred as well. With a focus on the reduction of preventable deaths, the data underscore the importance of an emergency transport system as a critical component of a community distribution system.

The current misoprostol distribution to the mother initiated by her onset of labor is fraught with delays, and needs to be critically re-appraised. Its alternative, advanced distribution, declined by community leaders, to avert its misuse for termination of pregnancy, requires reconsideration. Community leaders requested for documented evidence, or experience, that advance distribution of misoprostol could be implemented safely without abuse. This topic is a subject that is currently being explored.

The implementation of misoprostol distribution throughout Sokoto State benefitted from the careful use of the Ward Minimum Package on primary health care, to establish the state’s at-scale community health sub-system. This scale-up process also tapped into a range of negotiations to secure the trust and agreement of communities and their gatekeepers in the scale-up of and adoption of health interventions. The sensitivities associated with the use of misoprostol are fragile and significant in a conservative Islamic society. Not to have addressed key issues up front in the program design phase with the right sensitives could have caused an outright community refusal of a highly efficacious, lifesaving intervention. The government-led dialogue with community leaders, which lasted for six months ahead of program commencement, helped communities attain a state of readiness to adopt the intervention. It was also crucial that WDC chairmen acted as a rapid response team that respectfully and thoroughly addressed opposition or misinformation when it arose.

Our overall approach to scale up of misoprostol was largely consistent with factors associated with successful scale up outlined in a framework by Simmons, *et al*.[34] Their framework identified eleven characteristics such as “clear messages through which the advantages of the innovation are made visible,” “adaptation of innovation to local context,” “strong diffusion channels,” and “systematic use of evidence on the process and outcomes of scaling up.” In our context, all 11 characteristics were adequately satisfied. The constraint in scale up was not in the adoption of misoprostol. Rather, it was in the failure in the procurement of supplies to meet demand. Simmons, *et al*. highlighted the need for program scale up efforts to balance demand with supply to ensure quality. In our context, where the government made commitments to finance the regular procurement of misoprostol, not doing so in the face of evidence-related forecasts, suggests gaps in governance. The Simmons, *et al*. framework did not address governance-related lack of performance in program scale-up.

Although Spicer, *et al*. have suggested that scaling up is largely a craft and not a science, our experience provides evidence that securing government and community support is a critical component in bringing programs to scale. [35] Government officials had insisted on verifiable evidence as proof of value added and for use to advocate for additional financing. Investing in sound, simple, scientific methods to track primary health care progress, and sharing the findings with decision makers in real time is vital. In our experience this aided dialogue on program sustainability.

While successful community-based misoprostol has highlighted the importance of continuing to provide community-based solutions to improve maternal outcomes, such programs will not by themselves end all preventable maternal deaths. The verbal autopsy findings also underscored the need to continue to advocate for improved emergency transport systems and their use, and improved quality of care in facility-based deliveries for all mothers.

Notwithstanding the focus on misoprostol in this paper, we strongly advocate the integrated approach to the distribution of misoprostol with chlorhexidine digluconate 7.1% gel to mother-newborn dyads. Such program integration requires that community leaders’ concerns about misoprostol are anticipated and tackled upfront. We posit that such concerns can be expected to emerge in settings with social norms similar to that of Sokoto State. In this particular setting, the invention of the CDK—a solution that gave communities control of medicines supplies—and permitted scale up, also created problems of harmful delays of medicine delivery to homes. Program managers also need to be prepared to work with communities, using evidence, to understand the unintended consequences of their own program conditions. Although chlorhexidine digluconate 7.1% gel proved not to be controversial, not to have entered into trust-based community dialogue on misoprostol, could also have put chlorhexidine at risk of being rejected as well. In environments beset with fragile trust and high maternal mortality ratios, our documentation of the case of misoprostol will hopefully heighten the need for a keener awareness of such sensitivities by program managers.

## Study limitations

The program was designed to implement an intervention at scale to distribute a medicine of proven efficacy. As such, control or counterfactual groups to establish program effect were neither warranted nor of added value. Given that our data were collected in the context of a program setting that was not controlled, it is possible that the CDKs responsible for the data collection may have entered false data, suffered recall bias, kept incomplete records or did not report maternal deaths. The triangulation of information sources, between the CDK, CBHV and facility in-charges, the double data entry and the data quality checks that were undertaken substantially reduced the possibility of bias and incomplete records. To the best of our understanding, there was no reason for CDKs or the CBHVs to intentionally misstate data collected. There were no discernible perverse incentives for them to report inaccurate numbers. All evidence indicates that the CDKs and CBHVs were effective and reliable data collectors. What this experience has shown is that the data necessary to measure impact of an intervention, implemented at scale, can be captured by trained community-based volunteers or workers. Finally, we would argue that in the development of community-based data collection tools, particularly among low-literacy communities, efforts should be made to ensure the evaluation indicators chosen are very few in number, simple and strategic. Simple, clear data collection tools and easy-to-comprehend indicators will increase the probability of accurate documentation at the community level.

## Conclusion

To increase the likelihood that no woman dies of a preventable maternal death associated with PPH, community-based distribution of misoprostol in Sokoto State is a key intervention to increase access and use by underserved women. Securing appropriate quantities of misoprostol for community-based distribution requires sustained financial, political, and community support. Lessons learned from the Sokoto State scale up lend credence to the NPHCDA national strategy of the implementation of primary health through a village health worker system. In the end, the success and sustainability of primary health care interventions, implemented at scale, rests on whether impactful results were achieved equitably, and are well documented.

## Supporting information

S1 File Community-based outcome data collection form. (XLSX)

S2 File Data collection tool for verbal autopsies. (DOCX)

## Abbreviations

CBHV: Community-Based Health Volunteer
CDK: Community Drug Keeper
ICD: International Classification of Disease
IQR: Interquartile Range
JSI: JSI Research & Training Institute, Inc.
LGA: Local Government Areas
MNCH: Maternal, Newborn and Child Health
MMR: Maternal Mortality Ratio
MOLG: Ministry of Local Government
NAG/JNI: Nigeria Aid Group/Jama’atu Nasril Islam
NDHS: Nigeria Demographic and Health Survey
NPHCDA: National Primary Health Care Development Agency
NPC: National Population Commission
PHCUOR: Primary Health Care Under One Roof
PPH: Postpartum Haemorrhage
RDF: Revolving Drug Fund
SMOH: State Ministry of Health
SPHCDA: State Primary Health Care Development Agency
TSHIP: Targeted States High Impact Project
USAID: United States Agency for International Development
WDC: Ward Development Committee
WHO: World Health Organization

## References

[1] WHO. Trends in maternal mortality: 1990 to 2015 Estimates by WHO, UNICEF, UNFPA, World Bank Group and the United Nations Population Division [Internet]. 2015. Available: http://www.who.int/reproductivehealth/publications/monitoring/maternal-mortality-2015/en/

[2] Fapohunda BM, Orobaton NG. When women deliver with no one present in Nigeria: who, what, where and so what? PLoS One. 2013/08/13. 2013;8: e69569 10.1371/journal.pone.0069569 23936047 PMC3723888

[3] Fapohunda B, Orobaton N. Factors influencing the selection of delivery with no one present in Northern Nigeria: implications for policy and programs. Int J Womens Health. 2014/02/12. 2014;6: 171–183. 10.2147/IJWH.S54628 24516341 PMC3916635

[4] Austin A, Fapohunda B, Langer A, Orobaton N. Trends in delivery with no one present in Nigeria between 2003 and 2013. Int J Womens Health. Dove Press; 2015;7: 345–56. 10.2147/IJWH.S79573 25897265 PMC4396652

[5] Orobaton N, Austin A, Fapohunda B, Abegunde D, Omo K. Mapping the Prevalence and Sociodemographic Characteristics of Women Who Deliver Alone: Evidence From Demographic and Health Surveys From 80 Countries. Glob Heal Sci Pract. Johns Hopkins University- Global Health. Bloomberg School of Public Health, Center for Communication Programs; 2016;4: 99–113. 10.9745/GHSP-D-15-00261 PMC4807752 27016547

[6] Say L, Chou D, Gemmill A, Tunçalp Ö, Moller A-B, Daniels J, et al Global causes of maternal death: a WHO systematic analysis. Lancet Glob Heal. Elsevier; 2014;2: e323–e333. 10.1016/S2214-109X(14)70227-X 25103301

[7] WHO reccomendations for the prevention and treatment of postparutm haemorrhage [Internet]. 2012 [cited 4 May 2015]. Available: http://apps.who.int/iris/bitstream/10665/75411/1/9789241548502_eng.pdf

[8] Diop A, Daff B, Sow M, Blum J, Diagne M, Sloan NL, et al Oxytocin via Uniject (a prefilled single-use injection) versus oral misoprostol for prevention of postpartum haemorrhage at the community level: a cluster-randomised controlled trial. Lancet Glob Heal. Elsevier; 2016;4: e37–44. 10.1016/S2214-109X(15)00219-3 26718808

[9] Federal Republic of Nigeria: Essential Medicines List, 5th Revision [Internet]. 2010 [cited 5 May 2015]. Available: http://apps.who.int/medicinedocs/documents/s19018en/s19018en.pdf

[10] Jadesimi A, Okonofua FE. Tackling the unacceptable: Nigeria approves misoprostol for postpartum haemorrhage. J Fam Plann Reprod Health Care. British Medical Journal Publishing Group; 2006;32: 213–4. 10.1783/147118906778586660 17032506

[11] WHO Model List of Essential Medicines 17th Edition [Internet]. 2010 [cited 5 May 2015]. Available: http://whqlibdoc.who.int/hq/2011/a95053_eng.pdf?ua=1

[12] The Selection and Use of Essential Medicines: Report of the WHO Expert Committee, 2013 (including the 18th WHO Model List of Essential Medicines and the 4th WHO Model List of Essential Medicines for Children). In: WHO Technical Report Series 945 [Internet]. 2013 [cited 5 May 2015]. Available: http://apps.who.int/iris/bitstream/10665/112729/1/WHO_TRS_985_eng.pdf

[13] Winikoff B, Dabash R, Durocher J, Darwish E, Nguyen TNN, León W, et al Treatment of post-partum haemorrhage with sublingual misoprostol versus oxytocin in women not exposed to oxytocin during labour: a double-blind, randomised, non-inferiority trial. Lancet. 2010;375: 210–6. 10.1016/S0140-6736(09)61924-3 20060161

[14] Diadhiou M, Dieng T, Ortiz C, Mall I, Dione D, Sloan NL. Introduction of misoprostol for prevention of postpartum hemorrhage at the community level in Senegal. Int J Gynaecol Obstet. 2011;115: 251–5. 10.1016/j.ijgo.2011.08.002 21982859

[15] Ejembi C, Shittu O, Moran M, Adiri F, Oguntunde O, Saadatu B, et al Community-level distribution of misoprostol to prevent postpartum hemorrhage at home births in northern Nigeria. Afr J Reprod Health. 2014;18: 166–75. Available: http://www.ncbi.nlm.nih.gov/pubmed/25022154 25022154

[16] Prata N, Sreenivas A, Vahidnia F, Potts M. Saving maternal lives in resource-poor settings: facing reality. Health Policy. Elsevier; 2009;89: 131–48. 10.1016/j.healthpol.2008.05.007 18620778

[17] Raghavan S, Abbas D, Winikoff B. Misoprostol for prevention and treatment of postpartum hemorrhage: what do we know? What is next? Int J Gynaecol Obstet. 2012;119 Suppl: S35–8. 22883912 10.1016/j.ijgo.2012.03.013

[18] Weeks A, Ditai J, Ononge S, Durocher J, Faragher B, Byamugisha J, et al PL.03 Self-Administration of Misoprostol to Prevent Bleeding After Homebirths in Uganda: A Pilot Placebo-Controlled, Randomised Trial. Arch Dis Child—Fetal Neonatal Ed. 2013;98: A55–A55.

[19] Musa AO, Ijaiya MA, Saidu R, Aboyeji AP, Jimoh AA, Adesina KT, et al Double-blind randomized controlled trial comparing misoprostol and oxytocin for management of the third stage of labor in a Nigerian hospital. Int J Gynaecol Obstet. 2015; 10.1016/j.ijgo.2015.01.008 25835642

[20] Derman RJ, Kodkany BS, Goudar SS, Geller SE, Naik VA, Bellad MB, et al Oral misoprostol in preventing postpartum haemorrhage in resource-poor communities: a randomised controlled trial. Lancet. Elsevier; 2006;368: 1248–53. 10.1016/S0140-6736(06)69522-6 17027730

[21] WHO. Using lay health workers to improve access to key maternal and newborn health interventions in sexual and reproductive health [Internet]. 2013 [cited 5 May 2015]. Available: http://apps.who.int/iris/bitstream/10665/85617/1/WHO_RHR_13.09_eng.pdf?ua=1

[22] Orobaton N, Abegunde D, Shoretire K, Abdulazeez J, Fapohunda B, Lamiri G, et al A Report of At-Scale Distribution of Chlorhexidine Digluconate 7.1% Gel for Newborn Cord Care to 36,404 Newborns in Sokoto State, Nigeria: Initial Lessons Learned. Raju T, editor. PLoS One. Public Library of Science; 2015;10: e0134040 10.1371/journal.pone.0134040 26226017 PMC4520693

[23] Sokoto State Official Website:: About Sokoto State [Internet]. [cited 5 May 2015]. Available: http://www.sokotostate.gov.ng/aboutsokoto.php

[24] United Nations Fund for Population Activities–UNFPA–(2009) UNFPA supports traditional and religious leaders to mobilize for increased utilization of maternal health services in Sokoto State. Lagos, Nigeria:UNFP [Internet]. 2009 [cited 5 May 2015]. Available: http://nigeria.unfpa.org/sokoto.html

[25] National Population Commission [Nigeria] and ICF Macro. Nigeria Demographic and Health Survey 2008 [Internet]. Abuja, Nigeria. Abuja, Nigeria, and Rockville, Maryland, USA: NPC and ICF International; 2009 Available: http://www.dhsprogram.com/pubs/pdf/FR222/FR222.pdf

[26] National Population Commission (NPC) [Nigeria] and ICF International. Nigeria Demographic and Health Survey 2013 [Internet]. Abuja, Nigeria, and Rockville, Maryland, USA: NPC and ICF International; 2013 Available: http://dhsprogram.com/pubs/pdf/FR293/FR293.pdf

[27] Abegunde D, Kabo IA, Sambisa W, Akomolafe T, Orobaton N, Abdulkarim M, et al Availability, utilization, and quality of emergency obstetric care services in Bauchi State, Nigeria. Int J Gynaecol Obstet. 2015;128: 251–5. 10.1016/j.ijgo.2014.09.029 25497052

[28] Preventing newborn and maternal deaths in Nigeria [Internet]. [cited 6 May 2015]. Available: http://www.jsi.com/JSIInternet/Features/article/display.cfm?thisSection=Features&thisSectionTitle=Features&thisPage=stories&ctid=na&cid=na&tid=20&id=633

[29] JSI Research & Training Institute I. TARGETED STATES HIGH IMPACT PROJECT (TSHIP) Advancing Health in Bauchi and Sokoto States Annual Report PY5 [August 12, 2013—August 11, 2014] [Internet]. 2014. Available: http://pdf.usaid.gov/pdf_docs/PA00K9KZ.pdf

[30] Jegede AS. What led to the Nigerian boycott of the polio vaccination campaign? PLoS Med. 2007;4: 417–422. 10.1371/journal.pmed.0040073 PMC1831725 17388657

[31] Chen C. Rebellion against the polio vaccine in Nigeria: implications for humanitarian policy. African health sciences. 2004 pp. 205–207. 15687078 PMC2688336

[32] Ward minimum health care package, 2007–2012. [Abuja Nigeria: National Primary Health Care Development Agency; 2007.

[33] Cannon M, Charyeva Z, Oguntunde O, Sambisa W, Shoretire K, Orobaton N. A case study of community-based distribution and use of Misoprostol and Chlorhexidine in Sokoto State, Nigeria. Glob Public Health. 2016; 1–15. 10.1080/17441692.2016.1172102 27100376

[34] Simmons, Ruth1. Simmons R SJS up health service innovations: a framework for action. S up HSDFPI to PPGW 2007;1–30., Shiffman J. Scaling up health service innovations: a framework for action. Scaling up Heal Serv Deliv From Pilot Innov to Policies Program Geneva WHO. 2007; 1–30.

[35] Spicer N, Bhattacharya D, Dimka R, Fanta F, Mangham-Jefferies L, Schellenberg J, et al “Scaling-up is a craft not a science”: Catalysing scale-up of health innovations in Ethiopia, India and Nigeria. Soc Sci Med. 2014;121: 30–8. 10.1016/j.socscimed.2014.09.046 25306407

