# Supplementary material for: Implementing at-scale, community-based distribution of misoprostol tablets to mothers in the third stage of labor for the prevention of postpartum haemorrhage in Sokoto State, Nigeria: Early results and lessons learned
Source: PLoS One. 2017 Feb 24;12(2):e0170739. doi: 10.1371/journal.pone.0170739 (PMC5325195; doi:10.1371/journal.pone.0170739)
Supplement: S2 File — (DOCX) [file pone.0170739.s002.docx]

| **SOKOTO STATE MINISTRY OF LOCAL GOVERNMENT**  **(SUPPORTED BY USAID/TSHIP PROGRAMME)**  VERBAL AUTOPSY QUESTIONNAIRE FOR  MATERNAL DEATHS | **Female VA Form No.** | FORMNO |
| --- | --- | --- |

Instructions to interviewer: Introduce yourself and explain the purpose of your visit. Ask to speak to the mother or to another adult caretaker who was present during the illness that led to death. If this is not possible, arrange a time to revisit the compound when the mother or caretaker will be home. Before interviewing the person, explain to him/her that participation in the interview is voluntary; s/he can refuse to answer any question and s/he can stop the interview at any time. Explain to him/her that the information provided is only for research purposes and will be confidential.

1. **BACKGROUND:**

| 1.1. LGA name & Code…………………..……………………………………………..….. |  |  |  | Ward |
| --- | --- | --- | --- | --- |
| 1.2. Ward Name & Code………………………………………………………………… |  |  |  | LGA |
| 1.3. Address ……………………………………………………………………………………………………………ADDRESS | | | | |
| 1.4. Drug keeper Name…………………………………………………………………………………………….…..DKEEPER | | | | |
| 1.5. Deceased woman’s name………………………………………………………………………………………WOMNAME | | | | |

| 1.6 Deceased woman’s age:………………………………………………………………………….. |  |  | AGE |
| --- | --- | --- | --- |

| 1.7 Age group:……………………………………………. | 1. 15 –19 yrs | 2. 20 – 45 yrs | 3. 45+ yrs | AGEGRP |
| --- | --- | --- | --- | --- |

| 1.8 Date of death: ………………………………………...…….. |  |  |  |  |  |  | DATEDIED |
| --- | --- | --- | --- | --- | --- | --- | --- |

| 1.9 Date of visit: …………………………………………………….. |  |  |  |  |  |  | DATEVISIT |
| --- | --- | --- | --- | --- | --- | --- | --- |
| 1.10 Staff code: ………………………………………….………………….…………………. | | | | |  |  | FW |

| 1.11 Is a respondent available?...................................................................................... | | 1. Yes | 2. No | | | RESPOND |
| --- | --- | --- | --- | --- | --- | --- |
| 1.12 What is the respondent’s name?..... |  | | | | | RESNAME |
| 1.13 Respondent’s age……………………………………………………………………….. | | | |  |  | RESPAGE |

1.14 What is your relation to the deceased? Are you her husband, mother etc…?

| 11. Husband | 12. Mother | 13. Sister | 14. Mother-in-law | RELATION |
| --- | --- | --- | --- | --- |
| 15. Sister-in-law | 16. Friend | 17. Daughter | 18. Son |  |
| 19. Brother | 20. Brother-in-law | 21. Father | 22. Father-in-law |  |
| 23. Uncle | 24. Aunt | 25. Other: | |  |

**2. CIRCUMSTANCES SURROUNDING HER DEATH:**

| 2.1 Where did she die? | 1. Clinic/hospital or  maternity home | 2. On way to hospital/clinic | 3. At TBA/healer’s home, or  spiritualist | PLACEDIE |
| --- | --- | --- | --- | --- |
|  | 4. At home | 5. Other (Specify): | |  |

| 2.2 IF THE ANSWER TO 2.1 IS 1, STATE WHERE. ……………………………………..……………………………………………. | HOSPITAL |
| --- | --- |

| 2.3 IF SHE DIED OUTSIDE THE HOME:  Was she conscious when she arrived at where she died?.............. | 1. Yes | 2. No | 8. NK | 9. NA | COMADIE |
| --- | --- | --- | --- | --- | --- |

| 2.4 Were you present at the time she died?............................................................................ | 1. Yes | 2. No | ATDEATH |
| --- | --- | --- | --- |
| 2.5 Were you present when her condition started to deteriorate?.......................................... | 1. Yes | 2. No | ATWORSE |
| 2.6 Did you care for her in the final illness/period leading to her death?............................... | 1. Yes | 2. No | CARED |

**2.7 TYPE OF DEATH**

*These questions are extremely important. We may not know from the monthly visits that the woman was pregnant, especially if she was early on in her pregnancy. Please ask these questions carefully and sensitively to make sure that we are certain of the pregnancy status of the woman when she died.*

| 2.7.1 Was she pregnant when she died?............................................. | | 1. Yes | 2. No | 8. NK | PREGDIE |
| --- | --- | --- | --- | --- | --- |
| 2.7.1.1 | If so, what month in pregnancy did she die?  99 = Not applicable; did not die in pregnancy………………………… | |  |  | PMTHDIE |

| 2.7.2 | Did she die during labour or delivery?...................................... | 1. Yes | 2. No | 8. NK | LABDIE |
| --- | --- | --- | --- | --- | --- |
| 2.7.2.1 | At what month did the pregnancy end?  99 = Not applicable; was still pregnant at the time she died…………….. | |  |  | PREGEND |

| 2.7.3 | Did she die soon after delivery, that is, within 24 hours?......... | 1. Yes | 2. No | 8. NK | DELIVDIE |
| --- | --- | --- | --- | --- | --- |
| 2.7.4 | Did she die within the 42 days after delivery?.......................... | 1. Yes | 2. No | 8. NK | PPDIE |
| 2.7.4.1 | How many days after delivery did she die?  [99 = NA]……………………...………………………………………… | |  |  | PPDAYS |

| 2.7.5 | Was she nursing an infant at the time of her death, that is, did she have a baby who was less than 12 months of age?............. | 1. Yes | 2. No | 8. NK | BFEEDDIE |
| --- | --- | --- | --- | --- | --- |

| 2.8 Did she want this pregnancy?..................................................... | 1. Yes | 2. No | 8. NK | 9. NA | LIKEPREG |
| --- | --- | --- | --- | --- | --- |
| 2.9 Do you think she did anything to make the pregnancy end?........ | 1. Yes | 2. No | 8. NK | 9. NA | TRYTOENDP |

**3. DETAILS OF EVENTS SURROUNDING HER DEATH (ADDITIONAL SPACE ON NEXT PAGE)**

3.1 *Write the details surrounding the death event from the beginning. Probe for what problems developed, who did*

*she see, where did she go, how was she treated. Also ask how much time it took from one intervention to the other.*

*If she was pregnant ask about events related to care in pregnancy and delivery and ask what happened to the baby.*

*If the informant says that they think she tried to do something to end the pregnancy, please ask them to elaborate here.*

*You can carefully probe for this by asking “did she do anything to bring back her menses? For example, did she use*

*enema, herbs, or drug store medicine?”*

|  |  |  |
| --- | --- | --- |
|  |  |  |
|  |  |  |
|  |  |  |
|  |  |  |
|  |  |  |
|  |  |  |
|  |  |  |
|  |  |  |
|  |  |  |
|  |  |  |
|  |  |  |
|  |  |  |
|  |  |  |
|  |  |  |
|  |  |  |
|  |  |  |
|  |  |  |
|  |  |  |
|  |  |  |
|  |  |  |
|  |  |  |
|  |  |  |
|  |  |  |
|  |  |  |
| *NOTE FOR SUPERVISOR: Please write in the space below if you suspect that this woman may have had an abortion and, if yes, the reason why you suspect this. You should also confer with the fieldworker from this area if necessary.* | | |
|  |  |  |
|  |  |  |
|  |  |  |

**3.2 SUMMARY OF SIGNS AND SYMPTOMS REPORTED BY RESPONDENT**

| Symptoms | Day since start of illness | Duration  (days) | Severity  Mild/Moderate=1  Severe=2 |
| --- | --- | --- | --- |
| 3.2.1 |  |  |  |
| 3.2.2 |  |  |  |
| 3.2.3 |  |  |  |
| 3.2.4 |  |  |  |
| 3.2.5 |  |  |  |
| 3.2.6 |  |  |  |
| 3.2.7 |  |  |  |
| 3.2.8 |  |  |  |
| 3.2.9 |  |  |  |
| 3.2.10 |  |  |  |

**3.3 LIST OF HOSPITAL ADMISSIONS IN THE PAST 2 YEARS (BEGIN WITH MOST RECENT)**

| Name of health facility | Date (day/month/year) | Reasons for hospitalisation |
| --- | --- | --- |
| 3.3.1 | / / |  |
| 3.3.2 | / / |  |
| 3.3.3 | / / |  |
| 3.3.4 | / / |  |
| 3.3.5 | / / |  |

**3.4 INJURIES**

| 3.4 Did she sustain any injury which led to her death?................... | 1. Yes | 2. No | 8. NK | INJURY |
| --- | --- | --- | --- | --- |

3.4.1 If yes, what kind of injury or accident? (*Allow respondent to answer spontaneously*)

| 1. Transport accident  (pedestrian) | 2. Transport accident  (passenger/driver) | 3. Fall | 4. Drowning | INJTYPE |
| --- | --- | --- | --- | --- |
| 5. Poisoning | 6. Dog bite | 7. Snake bite | 8. Other animal bite or  sting |  |
| 9. Burn | 10. Firearm | 11. Sharp object  (e.g. knife) | 12. Circumcision |  |
| 13. Other assault or  abuse | 14. Other injury | 99. NA, no injury |  |  |

| Please specify if other bite, other assault or other injury….. |  | OTHINJ |
| --- | --- | --- |

| 3.4.2 Was the injury accidental or intentional?. | 1. Accidental | 2. Intentional | 8. NK | INJTY |
| --- | --- | --- | --- | --- |

| 3.4.3 Did she die at the site where the accident or injury occurred?........... | 1. Yes | 2. No | 8. NK | DSPOT |
| --- | --- | --- | --- | --- |

| 3.4.4 How long after the accident did she survive?...................... | 1<24 hours | 2.>24 hours | 8. NK | INJDU |
| --- | --- | --- | --- | --- |

| 3.4.5 After the accident, did she receive medical care before she died?..... | 1. Yes | 2. No | 8. NK | MDCARE |
| --- | --- | --- | --- | --- |

| 3.4.6 Do you think she may have done something to end her own life?..... | 1. Yes | 2. No | 8. NK | SUICIDE |
| --- | --- | --- | --- | --- |

| 3.4.6.1 If yes, how did she commit suicide? | | | | | |  |
| --- | --- | --- | --- | --- | --- | --- |
| 1. Hanging | 2. Poisoning | 3. Burns | 4. Gunshot | 5. Other (specify below) | 9. NA | HOWSUI |

| 3.4.6.2 If yes, why?.... |  | REASON |
| --- | --- | --- |

**3.5 DURATION OF ILLNESS**

| 3.5.1 For how long (in days) was she ill before she died? [ 88 = not known]............... |  |  | ILLDAYS |
| --- | --- | --- | --- |

| 3.5.2 Was she ill in the last month before her death (including before  an accident or injury)?....................................................................... | 1. Yes | 2. No | 8. NK | ILL |
| --- | --- | --- | --- | --- |

| 3.5.3 Was this a new illness, or something she had  frequently suffered from in the past?............... | 1. Yes,  new illness | 2. No,  old illness | 8. NK | 9. NA | NEWILL |
| --- | --- | --- | --- | --- | --- |

| 3.5.4 Did she die suddenly, or was it expected that  she was going to die?...................................... | 1. Yes,  suddenly | 2. No,  knew she would die | 8. NK | SUDDENDTH |
| --- | --- | --- | --- | --- |

**4. DEATH DURING PREGNANCY, LABOUR AND DELIVERY, OR AFTER A RECENT DELIVERY**

|  | COMPLETE THIS SECTION IF THE WOMAN DIED DURING PREGNANCY, LABOUR OR DELIVERY, OR IF SHE HAD  A DELIVERY OR ABORTION IN THE 6 WEEKS BEFORE SHE DIED  DEATH DURING PREGNANCY OR AFTER AN ABORTION: QUESTION 2.7.1 or 2.7.6 = Yes, COMPLETE ONLY SECTION 4.1  DEATH DURING LABOUR: QUESTION 2.7.2 = Yes, COMPLETE ONLY SECTIONS 4.1 AND 4.2  DEATH AFTER DELIVERY: QUESTION 2.7.3 = Yes, OR QUESTION 2.8.4 = Yes, COMPLETE THE WHOLE OF SECTION 4  (i.e. 4.1; 4.2 AND 4.3)  OTHERWISE DRAW A DOUBLE HORIZONTAL LINE THROUGH THIS SECTION AND PROCEED WITH SECTION 5. |
| --- | --- |

**4.1 NOW I’D LIKE TO ASK ABOUT PROBLEMS SHE MAY HAVE EXPERIENCED DURING THE PREGNANCY:**

| 4.1.1 How would you describe her health in general before  the pregnancy where she died?...................................... | 1. Excellent | 2. Good | 3. Poor | 8. NK | HEALTHY |
| --- | --- | --- | --- | --- | --- |

**Can you let me know if she experienced any of the following?**

| 4.1.2 Convulsions:……………………………………………………………….. | | 1. Yes | 2. No | | 8. NK | | FIT |  |
| --- | --- | --- | --- | --- | --- | --- | --- | --- |
| 4.1.2.1 At what month of pregnancy did this first occur? [88 = NK; 99 = NA]……………………. | | | |  | |  | FITPMTH | |
| 4.1.2.2 Did this also occur in the 7 days leading to her death?............. | 1. Yes | 2. No | 8. NK | | 9. NA | | FITPWKDTH |  |

| 4.1.3 Swelling of the face……………………………………………………….. | | 1. Yes | 2. No | | 8. NK | | FACE |  |
| --- | --- | --- | --- | --- | --- | --- | --- | --- |
| 4.1.3.1 At what month of pregnancy did this first occur? [88 = NK; 99 = NA]……………………. | | | |  | |  | FACEPMTH | |
| 4.1.3.2 Did this also occur in the 7 days leading to her death?............. | 1. Yes | 2. No | 8. NK | | 9. NA | | FACEWKDTH |  |

| 4.1.4 Swelling of the hands……………………………………………………... | | 1. Yes | 2. No | | 8. NK | | HAND |  |
| --- | --- | --- | --- | --- | --- | --- | --- | --- |
| 4.1.4.1 At what month of pregnancy did this first occur? [88 = NK; 99 = NA]……………………. | | | |  | |  | HANDPMTH | |
| 4.1.4.2 Did this also occur in the 7 days leading to her death?............. | 1. Yes | 2. No | 8. NK | | 9. NA | | HANDWKDTH |  |

| 4.1.5 Blurring of vision…………………………………………………………. | | 1. Yes | 2. No | | 8. NK | | BLUR |  |
| --- | --- | --- | --- | --- | --- | --- | --- | --- |
| 4.1.5.1 At what month of pregnancy did this first occur? [88 = NK; 99 = NA]……………………. | | | |  | |  | BLURPMTH | |
| 4.1.5.2 Did this also occur in the 7 days leading to her death?............. | 1. Yes | 2. No | 8. NK | | 9. NA | | BLURWKDTH |  |

| 4.1.6 Severe headache, to the degree that she was not able to work…………….. | | 1. Yes | 2. No | | 8. NK | | HEAD |  |
| --- | --- | --- | --- | --- | --- | --- | --- | --- |
| 4.1.6.1 At what month of pregnancy did this first occur? [88 = NK; 99 = NA]……………………. | | | |  | |  | HEADPMTH | |
| 4.1.6.2 Did this also occur in the 7 days leading to her death?............. | 1. Yes | 2. No | 8. NK | | 9. NA | | HEADWKDTH |  |

| 4.1.7 Doctor or nurse said she had “eclampsia” or severe hypertension:………. | | 1. Yes | 2. No | | 8. NK | | ECLAMP |  |
| --- | --- | --- | --- | --- | --- | --- | --- | --- |
| 4.1.7.1 At what month of pregnancy did this first occur? [88 = NK; 99 = NA]……………………. | | | |  | |  | ECLAMPPMTH | |
| 4.1.7.2 Did this also occur in the 7 days leading to her death?............. | 1. Yes | 2. No | 8. NK | | 9. NA | | ECLAMPWKDT |  |

| 4.1.8 Bleeding in pregnancy?................................................................................. | | 1. Yes | 2. No | | 8. NK | | APH |  |
| --- | --- | --- | --- | --- | --- | --- | --- | --- |
| 4.1.8.1 At what month of pregnancy did this first occur? [88 = NK; 99 = NA]……………………. | | | |  | |  | APHPMTH | |
| 4.1.8.2 Did this also occur in the 7 days leading to her death?............. | 1. Yes | 2. No | 8. NK | | 9. NA | | APHWKDTH |  |

| 4.1.9 Abdominal pain with bleeding?.................................................................... | | 1. Yes | 2. No | | 8. NK | | ABDPAIN |  |
| --- | --- | --- | --- | --- | --- | --- | --- | --- |
| 4.1.9.1 At what month of pregnancy did this first occur? [88 = NK; 99 = NA]……………………. | | | |  | |  | ABDPAINMTH | |
| 4.1.9.2 Did this also occur in the 7 days leading to her death?............. | 1. Yes | 2. No | 8. NK | | 9. NA | | ABDPAINWK |  |

| 4.1.10 Did she complain that she could not feel the baby move?.......................... | | 1. Yes | 2. No | | 8. NK | | NOMOVE |  |
| --- | --- | --- | --- | --- | --- | --- | --- | --- |
| 4.1.10.1 At what month of pregnancy did this first occur? [88 = NK; 99 = NA]…………………... | | | |  | |  | NOMOVEMTH | |
| 4.1.10.2 Did this also occur in the 7 days leading to her death?........... | 1. Yes | 2. No | 8. NK | | 9. NA | | NOMOVEWK |  |

| 4.1.11 Severe and continuous abdominal pain that was not labour pain?............. | | 1. Yes | 2. No | | 8. NK | | LONGPAIN |  |  |
| --- | --- | --- | --- | --- | --- | --- | --- | --- | --- |
| 4.1.11.1 At what month of pregnancy did this first occur? [88 = NK; 99 = NA]…………………... | | | |  | |  | LONGPAINMT | |  |
| 4.1.11.2 Did this also occur in the 7 days leading to her death?........... | 1. Yes | 2. No | 8. NK | | 9. NA | | LONGPAINWK | | |

| 4.1.12 Foul smelling vaginal discharge in pregnancy?.......................................... | | 1. Yes | 2. No | | 8. NK | | DISCHARGE |  |
| --- | --- | --- | --- | --- | --- | --- | --- | --- |
| 4.1.12.1 At what month of pregnancy did this first occur? [88 = NK; 99 = NA]…………………... | | | |  | |  | DISCHPMTH | |
| 4.1.12.2 Did this also occur in the 7 days leading to her death?........... | 1. Yes | 2. No | 8. NK | | 9. NA | | DISCHWKDTH |  |

| 4.1.13 Very hot fever at any time during pregnancy?............................................ | | 1. Yes | 2. No | | 8. NK | | HFEVER |  |  |
| --- | --- | --- | --- | --- | --- | --- | --- | --- | --- |
| 4.1.13.1 At what month of pregnancy did this first occur? [88 = NK; 99 = NA]…………………... | | | |  | |  | HFEVERPMTH | |  |
| 4.1.13.2 Did this also occur in the 7 days leading to her death?........... | 1. Yes | 2. No | 8. NK | | 9. NA | | HFEVERWKDT | | |

| 4.1.14 Eyes became yellow?................................................................................. | | 1. Yes | 2. No | | 8. NK | | JAUNDICE |  |
| --- | --- | --- | --- | --- | --- | --- | --- | --- |
| 4.1.14.1 At what month of pregnancy did this first occur? [88 = NK; 99 = NA]…………………... | | | |  | |  | JAUNPMTH | |
| 4.1.14.2 Did this also occur in the 7 days leading to her death?........... | 1. Yes | 2. No | 8. NK | | 9. NA | | JAUNWKDTH |  |

| 4.1.15 Urine became dark like coca cola……………………………………….. | | 1. Yes | 2. No | | 8. NK | | COKEPISS |  |
| --- | --- | --- | --- | --- | --- | --- | --- | --- |
| 4.1.15.1 At what month of pregnancy did this first occur? [88 = NK; 99 = NA]…………………... | | | |  | |  | COKEPISMTH | |
| 4.1.15.2 Did this also occur in the 7 days leading to her death?........... | 1. Yes | 2. No | 8. NK | | 9. NA | | COKEPISWKDT |  |

| 4.1.16 Did a doctor examine her blood and told her she was short of blood?....... | | 1. Yes | 2. No | | 8. NK | | ANEMIA |  |
| --- | --- | --- | --- | --- | --- | --- | --- | --- |
| 4.1.16.1 At what month of pregnancy did this first occur? [88 = NK; 99 = NA]…………………... | | | |  | |  | ANEMIAPMTH | |
| 4.1.16.2 Did this also occur in the 7 days leading to her death?........... | 1. Yes | 2. No | 8. NK | | 9. NA | | ANEMIWKDTH |  |

| 4.1.17 Did she have palpitations and shortness of breath?.................................... | | 1. Yes | 2. No | | 8. NK | | PALP |  |
| --- | --- | --- | --- | --- | --- | --- | --- | --- |
| 4.1.17.1 At what month of pregnancy did this first occur? [88 = NK; 99 = NA]…………………... | | | |  | |  | PALPPMTH | |
| 4.1.17.2 Did this also occur in the 7 days leading to her death?........... | 1. Yes | 2. No | 8. NK | | 9. NA | | PALPWKDTH |  |

| 4.1.18 Did she have any other serious problem during the pregnancy?................ | | 1. Yes | 2. No | 8. NK | | OPREGPROB |  |
| --- | --- | --- | --- | --- | --- | --- | --- |
| 4.1.19 What? |  | | | | SERIOUS PRB | | |

**4.2 DEATH DURING LABOUR, DELIVERY, OR UP TO 6 WEEKS AFTER DELIVERY**

IF SHE DIED DURING PREGNANCY, THAT IS, BEFORE THE ONSET OF LABOUR, DRAW A DOUBLE LINE

THROUGH SECTIONS 4.2 AND 4.3 AND CONTINUE WITH SECTION 5.1

| 4.2.1 Did the waters break before labour  or during labour?................................. | 1. Before labour started | 2. During labour | 8. Don’t know | WATERBRK |
| --- | --- | --- | --- | --- |

4.2.2 How much time before she started labour did the waters break?

|  | 1.Less than 4 hours | 2. 4 to 24 hours | 3. More than 24 hours | 8. Don’t know | 9. NA, broke during labour | TIMEBRK |
| --- | --- | --- | --- | --- | --- | --- |

| 4.2.3 How long was it from when she started labour pains till she delivered (or died)?  [DAYS:HOURS]…………………………………………………………………….. |  | **:** |  |  | TIMELAB |
| --- | --- | --- | --- | --- | --- |
| 4.2.4 How long was it from when she started strong and regular labour pains till she  delivered or died? [DAYS:HOURS]………………………………………………… |  | **:** |  |  | LONGLAB |

| 4.2.5 Did anyone give her any herbs or drugs to encourage labour?..................... | 1. Yes | 2. No | 8. NK | AUGMENT |
| --- | --- | --- | --- | --- |
| 4.2.6 Did they put any IV drip before the delivery or before she died?................ | 1. Yes | 2. No | 8. NK | IVDRIP |

4.2.7 In the opinion of the most experienced person who was around:

| 4.2.7.1 Was the length of labour short,  normal or long?....................... | 1. Short | 2. Normal | 3. Too long | 8. NK | 9. NA, died | TOOLONG |
| --- | --- | --- | --- | --- | --- | --- |
| 4.2.7.2 Was the labour difficult?................................. | | 1. Yes | 2. No | 8. NK | 9. NA, died | DIFFICULT |

| 4.2.8 How many babies did she delivered?...................................................................................... |  |  | NUMBABIES |
| --- | --- | --- | --- |

4.2.9 What happened to the first baby?

|  | 1. (term live birth) | 2 Term stillbirth | 3. Live birth, but died | BABY1 |
| --- | --- | --- | --- | --- |
|  | 4. premature, lost the baby | 5. ectopic | 6. Lost before 6mo |  |

4.2.10 What happened to the second baby?

|  | 1. Term live birth | 2. Term stillbirth | | 3. Live birth, but died | | BABY2 |
| --- | --- | --- | --- | --- | --- | --- |
|  | 4. Premature, lost the baby | 5. ectopic | 6. Lost before 6mo | | 9. NA, one child |  |

4.2.11 What happened to the third baby?

|  | 1. Term live birth | 2. Term stillbirth | | 3. Live birth, but died | | BABY3 |
| --- | --- | --- | --- | --- | --- | --- |
|  | 4. Premature, lost the baby | 5. ectopic | 6. Lost before 6mo | | 9. NA, one child |  |

| 4.2.12 Where did she give birth? | 1. Clinic or hospital | 2. Private maternity home | 3. At home/TBA | PLACEBIRTH |
| --- | --- | --- | --- | --- |
|  | 4. Other:  …………………………. | 5. On the way to hospital | 8. NK |  |

| 4.2.13 IF THE ANSWER IS 1 OR 2, STATE WHERE. ………………………………………...……………… | HOSPITAL2 |
| --- | --- |

| 4.2.14 Who delivered  the baby?......... | 1. Doctor | 2. Midwife | 3. TBA | 4.Other  person/relative | 5. Delivered herself  Alone (No one) | 8. NK | WHODELIV |
| --- | --- | --- | --- | --- | --- | --- | --- |

| 4.2.15 Did she have a delivery  through the vagina?............. | 1.Normally, through  the vagina | 2. Baby was pulled with  an instrument | 3. By CS | 8. NK | TYPEDELIV |
| --- | --- | --- | --- | --- | --- |

| 4.2.16 Which part of the baby came out first?............... | 1. Head | | 2. Feet/bottom | | | 3. CS | | 8. NK | POSITION |  |
| --- | --- | --- | --- | --- | --- | --- | --- | --- | --- | --- |
| 4.2.17 Did she know she was going to have a CS before she went  into labour?................................................................................ | | 1. Yes | | 2. No | 8. NK | | 9. NA; no CS | | KNOWCS | |

4.2.18 What made the doctor decide to do a CS?

|  | 1. Bleeding during  pregnancy (APH) | 2. Obstructed labour | 3. Previous CS | | 4. Toxaemia | | WHYCS |
| --- | --- | --- | --- | --- | --- | --- | --- |
|  | 5. Malpresentation | 6. Other: | | 8. NK | | 9. NA; no CS |  |

| 4.2.19 Did the placenta come out on its own?....................................................... | 1. Yes | 2. No | 8. NK | PLACENTA |
| --- | --- | --- | --- | --- |

| 4.2.20 How long after the baby came out did the placenta come out? (in hours)………….. |  |  |  | TIMESTAGE3 |
| --- | --- | --- | --- | --- |

| 4.2.121 Did someone have to put his/her hand inside her womb to remove  the placenta?.............................................................................................. | 1. Yes | 2. No | 8. NK | MRP |
| --- | --- | --- | --- | --- |

| 4.2.22 Who  did this?.. | 1. Doctor | 2. Midwife | 3. TBA | 4.Other person/relative: | 8. NK | 9. NA | WHOREMOVE |
| --- | --- | --- | --- | --- | --- | --- | --- |

**Now I’d like to ask about problems she may have experienced during labour.**

# Can you let me know if she experienced any of the following?

| 4.2.23 Excessive bleeding during labour………………………………………... | 1. Yes | 2. No | 8. NK | LABBLEED |
| --- | --- | --- | --- | --- |
| 4.2.24 Convulsions during labour ………………………………………………. | 1. Yes | 2. No | 8. NK | LABFIT |
| 4.2.25 Fever during labour………………………………………………………. | 1. Yes | 2. No | 8. NK | LABFEVER |
| 4.2.26 Loss of consciousness during labour…………………………………….. | 1. Yes | 2. No | 8. NK | LABCOMA |
| 4.2.27 Burst or torn womb during delivery……………………………………… | 1. Yes | 2. No | 8. NK | RUPTURE |

**4.3 DEATH DURING DELIVERY OR UP TO 6 WEEKS AFTER DELIVERY**

IF SHE DIED DURING PREGNANCY OR LABOUR, DRAW DOUBLE HORIZONTAL LINE THROUGH THIS SECTION

**Now I’d like to ask about problems she may have experienced after delivery.**

# Can you let me know if she experienced any of the following?

| 4.3.1 Tear in the vagina after delivery………………………………………….. | 1. Yes | 2. No | 8. NK | TEARVAG |
| --- | --- | --- | --- | --- |

| 4.3.2 Heavy bleeding after delivery…………………………………. | | 1. Yes | 2. No | 8. NK | | PPHEM | |
| --- | --- | --- | --- | --- | --- | --- | --- |
|  | 4.3.2.1 How many days after delivery did this occur?............................ | | |  |  | WHENPPHEM |  |
|  | 4.3.2.2 How many days did it last for?.................................................... | | |  |  | DAYSPPHEM |  |

| 4.3.3 Convulsions after delivery…………………………………… | | 1. Yes | 2. No | 8. NK | | PPFIT | |
| --- | --- | --- | --- | --- | --- | --- | --- |
|  | 4.3.3.1 How many days after delivery did this occur?............................ | | |  |  | WHENPPFIT |  |

| 4.3.4 Fever after delivery……………………………………………. | | 1. Yes | 2. No | 8. NK | | PPFEVER | |
| --- | --- | --- | --- | --- | --- | --- | --- |
|  | 4.3.4.1 How many days after delivery did this occur?............................ | | |  |  | WHNPPFEVER |  |
|  | 4.3.4.2 How many days did it last for?.................................................... | | |  |  | DAYSPPFEVE |  |

| 4.3.5 Loss of consciousness after delivery………………………….. | | 1. Yes | 2. No | 8. NK | | PPCOMA | |
| --- | --- | --- | --- | --- | --- | --- | --- |
|  | 4.3.5.1 How many days after delivery did this occur?............................ | | |  |  | WHENPPCOMA |  |
|  | 4.3.5.2 How many days did it last for?.................................................... | | |  |  | DAYSPPCOMA |  |

| 4.3.6 Foul discharge from the vagina………………………………... | | 1. Yes | 2. No | 8. NK | | PPDISCH | |
| --- | --- | --- | --- | --- | --- | --- | --- |
|  | 4.3.6.1 How many days after delivery did this occur?............................ | | |  |  | WHENPPDISC |  |
|  | 4.3.6.2 How many days did it last for?.................................................... | | |  |  | DAYSPPDISC |  |

| 4.3.7 Yellow eyes after delivery……………………………………... | | 1. Yes | 2. No | 8. NK | | PPJAUNDICE | |
| --- | --- | --- | --- | --- | --- | --- | --- |
|  | 4.3.7.1 How many days after delivery did this occur?............................ | | |  |  | WHPPJAUNDI |  |
|  | 4.3.7.2 How many days did it last for?.................................................... | | |  |  | DPPJAUNDI |  |

| 4.3.8 Urine dark like coca cola after delivery………………………... | | 1. Yes | 2. No | 8. NK | | PPCOKEPISS | |
| --- | --- | --- | --- | --- | --- | --- | --- |
|  | 4.3.8.1 How many days after delivery did this occur?............................ | | |  |  | WHPCOKEPIS |  |
|  | 4.3.8.2 How many days did it last for?.................................................... | | |  |  | DPPCOKEPIS |  |

| 4.3.9 Chest pain……………………………………………………… | | 1. Yes | 2. No | 8. NK | | PPCHESPAIN | |
| --- | --- | --- | --- | --- | --- | --- | --- |
|  | 4.3.9.1 How many days after delivery did this occur?............................ | | |  |  | WHPPCHESPA |  |
|  | 4.3.9.2 How many days did it last for?.................................................... | | |  |  | DPPCHESPAI |  |

| 4.3.10 Did she have any other problem  during labour or delivery?............ | 1. Yes (specify): | | 2. No | 8. NK | OLABDELPRO |  |
| --- | --- | --- | --- | --- | --- | --- |
| 4.3.10.1 When did this occur?..... | 1. During  labour | 2. After  delivery | 8. NK | 9. NA | WLABDELPRO | |

| 4.3.11 Was any operation done for her after she delivered?.................................. | 1. Yes | 2. No | 8. NK | PPOPS |
| --- | --- | --- | --- | --- |

| 4.3.11.1 What operation?......... | 1. Sewing her “under” | 2. Sewing her womb | 3. Removing her womb | | WHATOPS |
| --- | --- | --- | --- | --- | --- |
|  | 4. D and C or EOU | 5.Other: | 8. NK | 9. NA |  |

| 4.3.11.2 Who did this?.. | 1. Doctor | 2. Midwife/nurse | 4.Other: | 8. NK | 9. NA | WHOOPS |
| --- | --- | --- | --- | --- | --- | --- |

| 4.3.11.3 Where was this done? ………………………………………………………………………………….. | WHEREOPS |
| --- | --- |

| 4.3.11.4 Was she put to sleep for this operation?................................. | 1. Yes | 2. No | 8. NK | 9. NA | MAJOROPS |
| --- | --- | --- | --- | --- | --- |

**5. ADULT VERBAL AUTOPSY**

THE QUESTIONS IN SECTIONS 5.1 AND 5.2 ASK ABOUT THE OCCURRENCE AND DURATION OF SPECIFIC SYMPTOMS DURING THE TERMINAL ILLNESS. ENTER 90 IF DURATION IS 3 MONTHS AND ABOVE.

**Now I would like to check whether she had any of the following:**

**5.1 FEVER**

| 5.1.1 Did she have fever?............ | 1. Yes | 2. No | 8. NK |  | For how long? (in days)  [99=NA] |  |  | AFEVER  ADAYSFEVER |
| --- | --- | --- | --- | --- | --- | --- | --- | --- |

| 5.1.2 Was the fever…………………..…… | 1. Mild or  Moderate | 2. Extremely high | 8. NK | 9. NA,  no fever | SFE |
| --- | --- | --- | --- | --- | --- |

| 5.1.3 Was the fever continuous or on and  off?...................................................... | 1. Yes, continuous | 2. No, on and off | 8. NK | 9. NA,  no fever | ALONGFEV |
| --- | --- | --- | --- | --- | --- |

| 5.1.4 Did she have chills and/or rigors?.................................................. | 1. Yes | 2.No | 8. NK | RIG |
| --- | --- | --- | --- | --- |
| 5.1.5 Did she have night sweats?............................................................ | 1. Yes | 2. No | 8. NK | NCOU |

**5.2 HEART AND LUNG SYMPTOMS**

| 5.2.1 Did she have chest pain?... | 1. Yes | 2. No | 8. NK |  | For how long? (in days)  [99=NA] |  |  | ACHEST  ADCHEST |
| --- | --- | --- | --- | --- | --- | --- | --- | --- |

| 5.2.1.1 If yes, where was the  pain?................................ | 1. Over the  sternum | 2. Over the heart/  in the arm | 3. In the ribs | 9. NA, no  chest pain | WHEPAIN |
| --- | --- | --- | --- | --- | --- |

| 5.2.2 Did the pain start suddenly or  gradually?........................................... | 1. Yes, started  suddenly | 2. No, was gradual | 8. NK | 9. NA, no  chest pain | ASUDDENP |
| --- | --- | --- | --- | --- | --- |
| 5.2.3 When resting, was the pain…...……? | 1. Continuous | 2. On and off | 8. NK | 9. NA, no  chest pain | RESTPAIN |
| 5.2.4 During activity, was the pain………? | 1. Continuous | 2. On and off | 8. NK | 9. NA, no  chest pain | ACTPAIN |

| 5.2.5 When she had an attack of the pain,  how long did it last?......................... | 1. <30 min | 2. >30 min,  but <24 hrs | 3. >=24 hrs | 8. NK | 9. NA, no  chest pain | LASTCPAIN |
| --- | --- | --- | --- | --- | --- | --- |

| 5.2.6 Was she breathless on  light work?....................... | 1. Yes | 2. No | 8. NK |  | For how long? (in days)  [99=NA] |  |  | DYSP  ADAYSDYSP |
| --- | --- | --- | --- | --- | --- | --- | --- | --- |
| 5.2.7 Was she breathless on  lying flat?......................... | 1. Yes | 2. No | 8. NK |  | For how long? (in days)  [99=NA] |  |  | AORTHOPENA  ADAYSORTHO |
| 5.2.8 Did she have ankle  swelling?.......................... | 1. Yes | 2. No | 8. NK |  | For how long? (in days)  [99=NA] |  |  | AFEET  ADAYSFEET |
| 5.2.9 Did she have palpitations? | 1. Yes | 2. No | 8. NK |  | For how long? (in days)  [99=NA] |  |  | APALP  ADAYSPALP |
| 5.2.10 Did she look pale?........... | 1. Yes | 2. No | 8. NK |  | For how long? (in days)  [99=NA] |  |  | APALE  ADAYSPALE |
| 5.2.11 Did she have puffiness of  face?................................ | 1. Yes | 2. No | 8. NK |  | For how long? (in days)  [99=NA] |  |  | APUFFY  ADAYSPUFFY |

| 5.2.12 Did she have wheezing? | 1. Yes | 2. No | 8. NK |  | For how long? (in days)  [99=NA] |  |  | AWHEEZE  ADAYSWHEEZ |
| --- | --- | --- | --- | --- | --- | --- | --- | --- |
| 5.2.13 Did she have noisy  breathing?....................... | 1. Yes | 2. No | 8. NK |  | For how long? (in days)  [99=NA] |  |  | ANOISYBR  ADNOISYBR |
| 5.2.14 Did she have dry cough? | 1. Yes | 2. No | 8. NK |  | For how long? (in days)  [99=NA] |  |  | ADRYCOUGH  ADDRYCOUGH |
| 5.2.15 Did she have productive  cough?............................. | 1. Yes | 2. No | 8. NK |  | For how long? (in days)  [99=NA] |  |  | AWETCOUGH  ADWETCOUGH |
| 5.2.16 Was she coughing blood? | 1. Yes | 2. No | 8. NK |  | For how long? (in days)  [99=NA] |  |  | ACOUGHBLD  ADCOUGHBLD |

| 5.2.17 If she had a cough, when was it  worse?.............................................. | 1. Day | 2. Night | 3. Same | 8. NK | 9. NA | COUW |
| --- | --- | --- | --- | --- | --- | --- |

**5.3 APPETITE, WEIGHT LOSS AND SWALLOWING**

| 5.3.1 Did she have poor  appetite?............................ | 1. Yes | 2. No | 8. NK |  | For how long? (in days)  [99=NA] |  |  | ANOREXIA  ADNOREXIA |
| --- | --- | --- | --- | --- | --- | --- | --- | --- |
| 5.3.2 Did she have weight loss? | 1. Yes | 2. No | 8. NK |  | For how long? (in days)  [99=NA] |  |  | AWTLOSS  ADWTLOSS |

| 5.3.2.1 If she had weight loss, how long  did she have this before her death ? | 1. Days | 2. Months | 3. Years | 8.NK | 9. NA | DLOW |
| --- | --- | --- | --- | --- | --- | --- |

| 5.3.2.2 If she had weight loss, was it…… | 1. Mild/Moderate  (a little) | 2. Severe  (a lot) | 8. NK | 9. NA | SLW |
| --- | --- | --- | --- | --- | --- |

| 5.3.3 How did she look at the end of her  life?................................................... | 1. Normal | 2. Extremely thin  and wasted | 8. NK | 9. NA | WASTED |
| --- | --- | --- | --- | --- | --- |

| 5.3.4 Did she have mouth sores? | 1. Yes | 2. No | 8. NK |  | For how long? (in days)  [99=NA] |  |  | AMOUTH  ADMOUTH |
| --- | --- | --- | --- | --- | --- | --- | --- | --- |
| 5.3.5 Did she complain pain on  swallowing?....................... | 1. Yes | 2. No | 8. NK |  | For how long? (in days)  [99=NA] |  |  | ASWALLOW1  ADSWALLOW1 |
| 5.3.6 Did she have difficulty in  swallowing?....................... | 1. Yes | 2. No | 8. NK |  | For how long? (in days)  [99=NA] |  |  | ASWALLOW2  ADSWALLOW2 |

**5.4 ABDOMINAL SYMPTOMS**

| 5.4.1 Did she have abdominal  pain?.................................. | 1. Yes | 2. No | 8. NK |  | For how long? (in days)  [99=NA] |  |  | ABDPAIN  ADBDPAIN |
| --- | --- | --- | --- | --- | --- | --- | --- | --- |

| 5.4.1.1 What type of pain was it?...... | 1. Cramp | 2. Dull  ache | 3. Burning  pain | 4. Other | 8. NK | 9. NA | APAINTYPE |
| --- | --- | --- | --- | --- | --- | --- | --- |
| 5.4.1.2 Was the pain in upper, lower,  or all over her abdomen?....... | 1. Upper abdomen | 2. Lower abdomen | 3. All over abdomen | 4. Middle abdomen | 8. NK | 9. NA | TYPABDPAIN |
| 5.4.1.3 What was the severity of the  pain?...................................... | 1. Mild or moderate | | 2. Severe | | 8. NK | 9. NA | ABDPAINSEV |

| 5.4.2 Was she unable to pass  stool before her death?...... | 1. Yes | 2. No | 8. NK |  | For how long? (in days)  [99=NA] |  |  | NOSTOOLS  ADNOSTOOL |
| --- | --- | --- | --- | --- | --- | --- | --- | --- |

| 5.4.3 Did she have a mass in the  abdomen?.......................... | 1. Yes | 2. No | 8. NK |  | For how long? (in days)  [99=NA] |  |  | ABDMASS  ADBDMASS |
| --- | --- | --- | --- | --- | --- | --- | --- | --- |

| 5.4.4 Where exactly was  the mass?.................... | 1. Right upper abdomen | 2. Left upper abdomen | 3. Lower abdomen | 4. Other | 8. NK | 9. NA | SITEMASS |
| --- | --- | --- | --- | --- | --- | --- | --- |

| 5.4.5 Did she have abdominal  distension?......................... | 1. Yes | 2. No | 8. NK |  | For how long? (in days)  [99=NA] |  |  | ADISTEND  ADDISTEND |
| --- | --- | --- | --- | --- | --- | --- | --- | --- |

| 5.4.5.1 Did the distension start suddenly  (days) or gradually (over weeks)? | 1. Suddenly,  in days | 2. Gradually,  over weeks | 8. NK | 9. NA, no  distension | AFASTDIST |
| --- | --- | --- | --- | --- | --- |

| 5.4.6 Did her eye colour change  to yellow (jaundice)?......... | 1. Yes | 2. No | 8. NK |  | For how long? (in days)  [99=NA] |  |  | AJAUNDICE  ADJAUNDICE |
| --- | --- | --- | --- | --- | --- | --- | --- | --- |

**5.5 DIARRHOEA AND VOMITING**

| 5.5.1 Did she vomit?................... | 1. Yes | 2. No | 8. NK |  | For how long? (in days)  [99=NA] |  |  | AVOMIT  ADVOMIT |
| --- | --- | --- | --- | --- | --- | --- | --- | --- |

| 5.5.1.1 When the vomiting was most severe, how many times did she vomit in a day?  [88=NK; 99=NA]………………………………………………………………………………... |  |  | NUMVOM |
| --- | --- | --- | --- |

| 5.5.1.2 What did the vomit  look like?...................... | 1. Watery fluid | 2. Yellowish  fluid | | 3. Coffee  coloured fluid | 4. Blood | | VOMCONS |
| --- | --- | --- | --- | --- | --- | --- | --- |
|  | 5. Faecal matter | | 6. Other (specify) …… | | 8. NK | 9. NA |  |

| 5.5.2 Did she have diarrhoea?.... | 1. Yes | 2. No | 8. NK |  | For how long? (in days)  [99=NA] |  |  | ADIARRHOEA  ADDIARRHOE |
| --- | --- | --- | --- | --- | --- | --- | --- | --- |

| 5.5.2.1 Was the diarrhoea continuous?........ | 1. Yes | 2. No | 8. NK | 9. NA, no  diarrhoea | | DIARRCONT |
| --- | --- | --- | --- | --- | --- | --- |
| 5.5.2.2 What was the consistency of stools? | 1. Soft | 2. Watery | 8. NK | 9. NA, no  diarrhoea | | DIARRCONS |
| 5.5.2.3 When the diarrhoea was most severe, how many times did she pass stool in a day?  [88=NK; 99=NA]…………………………………………………………………………... | | | |  |  | NUMDIARR |

| 5.5.3 Did she have bloody  diarrhoea?.......................... | 1. Yes | 2. No | 8. NK |  | For how long? (in days)  [99=NA] |  |  | ADIARRBLD  ADDIARRBLD |
| --- | --- | --- | --- | --- | --- | --- | --- | --- |

| 5.5.4 Did she have sunken eyes? | 1. Yes | 2. No | 8. NK |  | For how long? (in days)  [99=NA] |  |  | ASUNKEYE  ADSUNKEYE |
| --- | --- | --- | --- | --- | --- | --- | --- | --- |

**5.6 URINARY SYMPTOMS**

| 5.6.1 Was there a change in the  colour of the urine?........... | 1. Yes | 2. No | 8. NK |  | For how long? (in days)  [99=NA] |  |  | AURINECOL  ADURINECOL |
| --- | --- | --- | --- | --- | --- | --- | --- | --- |

| 5.6.2 What was the colour of her  urine?.................................. | 1. Dark yellow | 2. Coffee-like | 3. Blood  stained | 8. NK | 9. NA | COLURINE |
| --- | --- | --- | --- | --- | --- | --- |

| 5.6.3 Did the amount of urine  she passed daily change?... | 1. Yes | 2. No | 8. NK |  | For how long? (in days)  [99=NA] |  |  | CQU  ADCQU |
| --- | --- | --- | --- | --- | --- | --- | --- | --- |

| 5.6.4 How much urine did she  pass in a day?..................... | 1. Too much | 2. Too little | 3. No urine at  all | 8. NK | 9. NA | AMOURINE |
| --- | --- | --- | --- | --- | --- | --- |

| 5.6.5 Did she have difficulty or  pain in passing urine?........ | 1. Yes | 2. No | 8. NK |  | For how long? (in days)  [99=NA] |  |  | DPU  ADDPU |
| --- | --- | --- | --- | --- | --- | --- | --- | --- |

| 5.6.5.1 What type of difficulty did she  have?................................................ | 1. Unable to  pass urine | 2. Continuous  dribbling | 3. Burningsensation | 4. Intensepain | DIFFURINE |
| --- | --- | --- | --- | --- | --- |
|  | 5. Other …………. | | 8. NK | 9. NA |  |

**5.7 NEUROLOGICAL SYMPTOMS**

| 5.7.1 Did she have headache?.... | 1. Yes | 2. No | 8. NK |  | For how long? (in days)  [99=NA] |  |  | AHEADACHE  ADHEADACHE |
| --- | --- | --- | --- | --- | --- | --- | --- | --- |

| 5.7.2 Did she become mentally  confused?........................... | 1. Yes | 2. No | 8. NK |  | For how long? (in days)  [99=NA] |  |  | ACONFUSE  ADCONFUSE |
| --- | --- | --- | --- | --- | --- | --- | --- | --- |
| 5.7.3 Did she have loss of  consciousness?.................. | 1. Yes | 2. No | 8. NK |  | For how long? (in days)  [99=NA] |  |  | ACOMA  ADCOMA |

| 5.7.4 Did she become confused or  unconscious suddenly or gradually?... | 1. Suddenly  (within hours) | 3. Within a day | 8. NK | AFASTCOMA |
| --- | --- | --- | --- | --- |
|  | 4. Slowly over a  few days | 5. Other ………. | 9. NA |  |

| 5.7.5 Was she paralysed on one  side of the body?............... | 1. Yes | 2. No | 8. NK |  | For how long? (in days)  [99=NA] |  |  | APARALYSE  ADPARALYSE |
| --- | --- | --- | --- | --- | --- | --- | --- | --- |
| 5.7.6 Did she have paralysis of  both legs?.......................... | 1. Yes | 2. No | 8. NK |  | For how long? (in days)  [99=NA] |  |  | ALEGDEAD  ADLEGDEAD |

| 5.7.7 How long did the paralysis take to develop?.. | 1. Instantly | 2. Hours | 3. Days | 4. Months | AFASTPAR |
| --- | --- | --- | --- | --- | --- |
|  | 5. Years | 8. NK | 9. NA |  |  |

| 5.7.8 Did she have neck pain?.... | 1. Yes | 2. No | 8. NK |  | For how long? (in days)  [99=NA] |  |  | ANEXKPAIN  ADNEXKPAIN |
| --- | --- | --- | --- | --- | --- | --- | --- | --- |
| 5.7.9 Did she have a stiff neck?. | 1. Yes | 2. No | 8. NK |  | For how long? (in days)  [99=NA] |  |  | ASTIFFNECK  ADSTIFFNEC |

| 5.7.10 Did she develop stiffness  of the whole body?.......... | 1. Yes | 2. No | 8. NK |  | For how long? (in days)  [99=NA] |  |  | ASTIFF  ADSTIFF |
| --- | --- | --- | --- | --- | --- | --- | --- | --- |

| 5.7.11 Did she have fits?............ | 1. Yes | 2. No | | 8. NK | |  | For how long? (in days)  [99=NA] | |  |  | AFIT  ADFIT |
| --- | --- | --- | --- | --- | --- | --- | --- | --- | --- | --- | --- |
| 5.7.11.1 Did she have stiffness  of the whole body during fits? | 1. Yes | 2. No | | 8. NK | |  | For how long? (in days)  [99=NA] | |  |  | ASTIFIT  ADSTIFIT |
| 5.7.11.2 When the fits were most frequent, how many did she have in a day?  [88=NK; 99=NA]…………………………………………………………………………... | | | | | | | | |  |  | NUMFITS |
| 5.7.11.3 Between fits, was she……………… | | | 1. Awake | | 2. Unconscious | | | 8. NK | 9. NA,  no fits | | BETFITS |
| 5.7.11.4 Did she have difficulty in opening  her mouth during fits?...................... | | | 1. Able to open | | 2. Unable to  open | | | 8. NK | 9. NA,  no fits | | MOUTHFITS |

| 5.7.12 Did she have pins and  needles in feet?................ | 1. Yes | 2. No | 8. NK |  | For how long? (in days)  [99=NA] |  |  | APINS  ADPINS |
| --- | --- | --- | --- | --- | --- | --- | --- | --- |

**5.8 RASHES, ULCERS AND SWELLINGS**

| 5.8.1 Did she have any rash?...... | 1. Yes | 2. No | 8. NK |  | For how long? (in days)  [99=NA] |  |  | ARASH  ADRASH |
| --- | --- | --- | --- | --- | --- | --- | --- | --- |

| 5.8.1.1 If yes, where was the rash?... | Face | 1. Yes | 2. No | 8. NK | 9. NA | RFACE |
| --- | --- | --- | --- | --- | --- | --- |
|  | Trunk | 1. Yes | 2. No | 8. NK | 9. NA | RSTRG |
|  | Extremities | 1. Yes | 2. No | 8. NK | 9. NA | REXTR |
|  | All over the body | 1. Yes | 2. No | 8. NK | 9. NA | RALLB |
|  | Other: (specify) | 1. Yes | 2. No | 8. NK | 9. NA | ROTHE |

| 5.8.1.2 If yes, what did the rash look like? | | | | | |  |
| --- | --- | --- | --- | --- | --- | --- |
| 1. Measles | 2. Rash with clear fluid | 3. Rash with pus | 4. Other (specify) | 8. NK | 9. NA | TRA |

| 5.8.1.3 Did the skin crack/split or peel after the rash started?..... | 1. Yes | 2. No | 8. NK | 9. NA | SKIRAS |
| --- | --- | --- | --- | --- | --- |

| 5.8.2 Did she have red eyes?.............................................................................. | 1. Yes | 2. No | 8. NK | SEY |
| --- | --- | --- | --- | --- |

| 5.8.3 Did she have itching of skin?.................................................................... | 1. Yes | 2. No | 8. NK | ITCH |
| --- | --- | --- | --- | --- |

| 5.8.4 Did she have ulcer or  swelling in breast?............. | 1. Yes | 2. No | 8. NK |  | For how long? (in days)  [99=NA] |  |  | ABREAST  ADBREAST |
| --- | --- | --- | --- | --- | --- | --- | --- | --- |

| 5.8.5 Did she have ulcer on any other part of the body?.................................... | | 1. Yes | 2. No | 8. NK | ULC |
| --- | --- | --- | --- | --- | --- |
| 5.8.5.1 If yes, please specify where the ulcer was... |  | | | 9. NA | ULCLOC |

| 5.8.6 Did she have swelling in  the neck?............................ | 1. Yes | 2. No | 8. NK |  | For how long? (in days)  [99=NA] |  |  | ANECKSW  ADNECKSW |
| --- | --- | --- | --- | --- | --- | --- | --- | --- |
| 5.8.7 Did she have swelling in  the armpit?......................... | 1. Yes | 2. No | 8. NK |  | For how long? (in days)  [99=NA] |  |  | APITSW  ADPITSW |
| 5.8.9 Did she have swelling in  the groin?........................... | 1. Yes | 2. No | 8. NK |  | For how long? (in days)  [99=NA] |  |  | AGROINSW  ADGROINSW |
| 5.8.10 Did she have swelling of  joints?.............................. | 1. Yes | 2. No | 8. NK |  | For how long? (in days)  [99=NA] |  |  | AJOINTS  ADJOINTS |

**5.9 ABNORMAL BLEEDING AND DISCHARGE**

| 5.9.1 Did she have bleeding from the body openings (other than her normal  menstruations)?......................................................................................... | 1. Yes | 2. No | 8. NK | BLEEO |
| --- | --- | --- | --- | --- |

| 5.9.2 Did she have abnormal  vaginal bleeding?.............. | 1. Yes | 2. No | 8. NK |  | For how long? (in days)  [99=NA] |  |  | AVAGBLEED  ADVAGBLEED |
| --- | --- | --- | --- | --- | --- | --- | --- | --- |
| 5.9.3 Did she have abnormal  vaginal discharge?............. | 1. Yes | 2. No | 8. NK |  | For how long? (in days)  [99=NA] |  |  | AVAGDISCH  ADVAGDISCH |

**5.10 MEDICAL CARE**

5.10.1 Had she been admitted to hospital for more than 2 days in the past 12 months?

|  | 1. Yes, for illness/complication  related to pregnancy or childbirth | 2. Yes,  for other illness | 3. Yes, for  accident/injury | 4. No | 8. NK | ADMISSION |
| --- | --- | --- | --- | --- | --- | --- |

| 5.10.2 Where was she admitted? …………………………………………………………………………... = NA WHERADM |
| --- |

| 5.10.3 Did she have any operation before death?................................................. | 1. Yes | 2. No | 8. NK | OPERATION |
| --- | --- | --- | --- | --- |
| 5.10.4 How many days before death did s/he have the operation? [88=NK; 99=NA]…..… | |  |  | OPDAYS |

| 5.10.5 If yes, what was the site of the operation?..................... | 1. Abdomen | 2. Heart | 3. Head | WHEREOP |
| --- | --- | --- | --- | --- |
|  | 4. Other | 8. NK | 9. NA |  |

Has a ‘doctor’ ever told her she had any of the following illnesses?

| 5.10.6 | Heart disease? | 1. Yes | 2 No | 8. NK | HEART |
| --- | --- | --- | --- | --- | --- |
| 5.10.7 | Hypertension? | 1. Yes | 2 No | 8. NK | HYPERTEN |
| 5.10.8 | Varicose veins? | 1. Yes | 2 No | 8. NK | VEINS |
| 5.10.9 | Kidney disease? | 1. Yes | 2 No | 8. NK | KIDNEY |
| 5.10.10 | Asthma? | 1. Yes | 2 No | 8. NK | ASTHMA |
| 5.10.11 | TB? | 1. Yes | 2 No | 8. NK | TB |
| 5.10.12 | Epilepsy? | 1. Yes | 2 No | 8. NK | EPILEPSY |
| 5.10.13 | Diabetes? | 1. Yes | 2 No | 8. NK | DIABETES |
| 5.10.14 | Jaundice or hepatitis? | 1. Yes | 2 No | 8. NK | JAUNDICE |
| 5.10.15 | Leprosy? | 1. Yes | 2 No | 8. NK | LEPROSY |
| 5.10.16 | Cancer? | 1. Yes | 2 No | 8. NK | CANCER |
|  | 5.10.16.1 If yes, please specify type: | | | | CANCTYP |
| 5.10.17 | HIV/AIDS? | 1. Yes | 2 No | 8. NK | HIVAIDS |
| 5.10.18 | Any other serious illness: | 1. Yes | 2 No | 8. NK | OTHILL |
| 5.10.18.1 If yes, please specify: ……………………. _____________________________________________ | | | | | OTHTYP |

| 5.10.19 Did she REGULARLY take any medicines for an illness or health  condition?.............................................................................................. | 1. Yes | 2. No | 8. NK | MEDICINE |
| --- | --- | --- | --- | --- |
| 5.10.20 Did she receive any drugs during her final illness?................................. | 1. Yes | 2. No | 8. NK | DRUGILL |
| 5.10.21 Did she receive any antibiotics during her final illness?......................... | 1. Yes | 2. No | 8. NK | ANTIBX |
| 5.10.22 Did she receive any anti-malarial drug during the illness?...................... | 1. Yes | 2. No | 8. NK | ANTIMAL |

| 5.10.22.1 What kind of antimalarial did she receive?...... | 1. ACTs | 2. Fansidar | 3. Quinine | | TYPMAL |
| --- | --- | --- | --- | --- | --- |
|  | 4. Amodiaquine | 5. Other | 8. NK | 9. NA |  |

**5.11 CAUSE OF DEATH**

| 5.11.1 Do you know the cause(s) of her death?.................................................... | 1. Yes | 2. No | 8. NK | RKC |
| --- | --- | --- | --- | --- |

5.11.2 What do you personally think was the cause of her death?

|  |  | YOUTHINK |
| --- | --- | --- |
|  |  |  |

5.11.3 Did anybody tell you the cause of her death? Who?

|  | 1. Yes, doctor | 2. Yes, nurse | 3. Yes, other  health worker | 4. Yes, other  health provider | 5. Other  person | 6. No | KNOWCAUSE |
| --- | --- | --- | --- | --- | --- | --- | --- |

5.11.4 What did they say it was?

|  |  | THEYSAY |
| --- | --- | --- |
|  |  |  |

5.11.5 Is there anything more concerning her death, which I have not asked about, that you want to tell me?

|  |  | TELLME |
| --- | --- | --- |
|  |  |  |

**6. SOCIO ECONOMIC CHARACTERISTICS**

**Now, I would like to ask some questions about her personal details and her household**

6.1 Did she attend school? What is the highest educational level that she reached?

| 1. None [CODE 99 FOR 6.2] | 2. Primary school | 3. Secondary Middle/continuation school, JSS | MEDLEV |
| --- | --- | --- | --- |
| 4. Technical/commercial/SSS  secondary school | 5. Post-middle college – teacher training, secretarial | 6. Post secondary – nursing, teacher, polytechnic, etc. |  |
| 7. University | 8. Not known |  |  |

| 6.2 Number of years successfully completed at the highest level reached [88 = NK]……………... |  |  | NUMYRS |
| --- | --- | --- | --- |

| 6.3 Was she married, widowed, divorced, or separated?.............................................................. | 1. Married | 2. Widowed | 3. Divorced | | MARRIED |
| --- | --- | --- | --- | --- | --- |
|  | 4. Separated | 5. Single, unmarried | | 8. NK |  |

6.4 What was her religion?

| 1. Christianity | 2. Islam | 5. Traditional African Religion | 6. Other: | 8. NK |  | RELIGION |
| --- | --- | --- | --- | --- | --- | --- |

| 6.5 Which ethnic  group did she belong to?...... | 1 Hausa/Fulani | 3. Yoruba | 4. Igbo | 5. Others……….. | ETHNIC |
| --- | --- | --- | --- | --- | --- |

| 6.6 Did she own any land?...................................................................................................... | 1. Yes | 2. No | WOWNLAND |
| --- | --- | --- | --- |

| 6.7 Did she have land to  farm on?.................... | 1. Yes,  her own | 2. Yes, part of  family land | 3. Yes part of  husband’s | 4. Yes, rented  land | 5. No | 8. NK | OWNLAND |
| --- | --- | --- | --- | --- | --- | --- | --- |

6.8 What did she grow on her land?

| 1. Food items, mainly for  home consumption | 2. Food items, mainly for  sale on the market | 3. Cash crops: yam, tobacco,  maize, tomatoes, etc. | 8. NK | 9. NA, no farm | CROPS |
| --- | --- | --- | --- | --- | --- |

6.9 Did she have a regular cash income/was she a salaried worker?

| 1. Yes, professional – teacher, nurse,  accounts, administrative | 2. Yes, clerical/secretarial | 3. Yes, seamstress, hairdresser etc. | | | SALARY |
| --- | --- | --- | --- | --- | --- |
| 4. Yes, trader/food seller | 5. Yes, labourer/domestic  Worker | 6. Other: | 7. No | 8. NK |  |

SAY NOW YOU ARE GOING TO ASK ABOUT HER ‘HOUSEHOLD’ AT THE TIME OF HER DEATH AND EXPLAIN WHAT A HOUSEHOLD IS

6.10. Who was the household head?

| 1. Her | 2. Her husband | 3. Her father | 4. Her mother | 5. Other: | 8. NK | HOUSEHEAD |
| --- | --- | --- | --- | --- | --- | --- |

| 6.11. In what year was the household head born? [88 = NK]…………………… | **1** | **9** |  |  | HHYOB |
| --- | --- | --- | --- | --- | --- |

| 6.12. How old is the household head now (in years)? [88 = NK]………………………………… |  |  | HHAGE |
| --- | --- | --- | --- |

6.13. What was the household head’s highest educational level reached?

| 1. None | 2. Primary school | 3.Middle,continua-tion school, JSS | 4. Technical, commercial, SSS, Secondary school | HHMEDLEV |
| --- | --- | --- | --- | --- |
| 5. Post-middle college, teacher training, secretarial | 6. Post-secondary, nursing, teacher, polytechnic | 7. University | 8. Not known |  |

| 6.14. What was the number of years that the household head completed at the highest level  reached? [88 = NK, 00 = no education]……… …………………….…………….………. |  |  | HHNUMYRS |
| --- | --- | --- | --- |

6.15. Did the household head have a regular cash income or salaried job?

| 1. Professional – teacher, nurse,  accounts, administrator etc. | 2. Clerical /  secretarial | 3. Trader / businessman /  driver with own car etc. | 4. Employed tradesman, driver  without own car, builder, etc. | | | HHSALARY |
| --- | --- | --- | --- | --- | --- | --- |
| 5. Farmer/labourer/domestic  worker | 6. Other: | | | 7. No | 8. NK |  |

| 6.16. Did members of the household do any farming?........................................................... | 1. Yes | 2. No | HHFARMING |
| --- | --- | --- | --- |
| 6.17. Did anyone in the household own any land?............................................................... | 1. Yes | 2. No | HHOWNLAND |
| 6.18. Did anyone in the household own their own farm?..................................................... | 1. Yes | 2. No | HHOWNFARM |

6.19. What did they grow?

| 1. Food items, mainly for  home consumption | 2. Food items, mainly for  sale on the market | 3. Cash crops – yam, tobacco,  maize, tomatoes, etc. | 9. NA, no farm | HHCROP |
| --- | --- | --- | --- | --- |

| 6.20. Did anyone in the household own:….. | Chickens or ducks? | 1. Yes | 2. No | CHICKEN |
| --- | --- | --- | --- | --- |
| 6.20.1 | Sheep or goats? | 1. Yes | 2. No | SHEEP |
| 6.20.2 | Other animals? | 1. Yes | 2. No | OTHANIM |
| 6.20.3 | Table? | 1. Yes | 2. No | TABLE |
| 6.20.4 | Sleeping mattress? | 1. Yes | 2. No | MATTRESS |
| 6.20.5 | Cupboard, wardrobe, room divider? | 1. Yes | 2. No | DIVIDER |
| 6.20.6 | Mosquito net? | 1. Yes | 2. No | MOSNET |
| 6.20.7 | Sewing machine? | 1. Yes | 2. No | SEWMACH |
| 6.20.8 | Bicycle? | 1. Yes | 2. No | BICYCLE |
| 6.20.9 | Radio? | 1. Yes | 2. No | RADIO |
| 6.20.10 | TV? | 1. Yes | 2. No | TV |
| 6.20.11 | Gas or electric cooker? | 1. Yes | 2. No | COOKER |
| 6.20.12 | Fridge or freezer? | 1. Yes | 2. No | FRIDGE |
| 6.20.13 | Motorcycle?.................................................. | 1. Yes | 2. No | MOTORCYCLE |
| 6.20.14 | Mobile phones | 1. Yes | 2. No | Mobile |
| 6.20.14a. How many mobile phones are there in this household | |  |  | Mobile_num |
| 6.20.15 | Car?............................................................... | 1. Yes | 2. No | CAR |

| 6.21. Did her household have electricity?............................................................................... | 1. Yes | 2. No | ELECTRIC |
| --- | --- | --- | --- |

6.22. What was the main source of drinking water for members of the household?

| 11. Piped into dwelling/yard/plot | 12. Public tap | 13. Handpump / closed bore hole | 14. Closed well | 15. Open well | WATER |
| --- | --- | --- | --- | --- | --- |
| 16. Stream / river | 17. Lake / dam /pond | 18. Water trucks | 19.Rain water | 20. Other |  |

6.23. How long did it take for her to go there, get water and come back?

| 1. Less than 15 minutes | 2. 15 minutes- less than 30 minutes | 3. 30 minutes – less than 60 minutes | REACH |
| --- | --- | --- | --- |
| 4. 60 minutes or more | 9. NA / drinking water source is in compound | |  |

6.24. What kind of toilet facility did the household have?

| 1. Flush latrine / WC | 2. Ventilated improved pit /VIP /KVIP | 3. Other pit latrine | 4. Open fields | DEFAEC |
| --- | --- | --- | --- | --- |
| 5. Defaecate in house, faeces transferred elsewhere / bucket latrine | | 6. Other: | |  |

| 6.25. What were the total number of rooms in the household used for sleeping? 88 = NK.............. |  |  | ROOMS |
| --- | --- | --- | --- |
| 6.26. What were the total number of people that slept in the household last night? 88 = NK........... |  |  | RESIDENT |

6.27. Do she own or rent the house she lived in, or did she have another type of arrangement, such as “perching”?

| 1. Sole Ownership | 2. Joint Ownership | 3. Renting | 4. Family/relation’s house | | OWNHOUSE |
| --- | --- | --- | --- | --- | --- |
| 5. House provided rent free | 6. Perching | 7. Other: | | 8. NK |  |

WHAT MATERIALS WERE USED IN THE CONSTRUCTION OF HER HOUSE [OBSERVE IF POSSIBLE]?

| 6.28. Floor of sleeping room | 1. Cement | 2. Mud/clay | 3. Other: | 8. NK | FLOORSLEEP |
| --- | --- | --- | --- | --- | --- |
| 6.29. Roofing | 1. Metal/asbestos | 2. Thatch/mud | 3. Other: | | ROOF |
| 6.30. Wall | 1. Cement | 2. Mud | 3. Other: | | WALL |

| 6.31. Did her household have a separate room with a roof just for cooking?........ | 1. Yes | 2. No | 8. NK | KITCHEN |
| --- | --- | --- | --- | --- |

| 6.32. Did her household have a separate sleeping room for children?.................. | 1. Yes | 2. No | 8. NK | SHARERM |
| --- | --- | --- | --- | --- |

| 6.33. Did her household have a domestic worker not related to the household  head?............................................................................................................. | 1. Yes | 2. No | 8. NK | DOMESTIC |
| --- | --- | --- | --- | --- |

**7. FERTILITY AND OBSTETRIC HISTORY**

**Now, I would like to ask you some questions about any pregnancies and children that she had.**

[IF SHE DIED DURING PREGNANCY, LABOUR , DELIVERY OR 42 DAYS AFTER DELIVERY, EXCLUDE THAT PREGNANCY OR BIRTH]

| 7.1 How many male children of her own did she have that lived with her when she died?  [00 = NONE] EXCLUDE ANY BIRTH FROM THE PREGNANCY WHICH LED TO HER DEATH……………….. |  |  | BOYALIVE1 |
| --- | --- | --- | --- |
| 7.2 How many male children of her own are living elsewhere? [00 = NONE]……………………………… |  |  | BOYALIVE2 |

| 7.3 How many female children of her own did she have that lived with her when she died?  [00 = NONE] EXCLUDE ANY BIRTH FROM THE PREGNANCY WHICH LED TO HER DEATH……………… |  |  | GIRLALIVE1 |
| --- | --- | --- | --- |
| 7.4 How many female children of her own are living elsewhere? [00 = NONE]………………………… |  |  | GIRLALIVE2 |

| 7.5 Did she have any children who were born alive but died later? How many?  [0 = NONE] EXCLUDE ANY BIRTH FROM THE PREGNANCY WHICH LED TO HER DEATH………………………… |  | DEADCHN |
| --- | --- | --- |
| 7.6 Did she ever lose a pregnancy? How many?  [0 = NONE] EXCLUDE THE PREGNANCY WHICH LED TO HER DEATH ……………………………………………….. |  | ABORTION |

| 7.7 Did she ever have a stillbirth? How many?  EXCLUDE ANY BIRTH FROM THE PREGNANCY WHICH LED TO HER DEATH……………………………………… |  | STIILBIRTH |
| --- | --- | --- |
| 7.8 Did she ever have an ectopic? How many?  [0 = NONE]. EXCLUDE THE PREGNANCY WHICH LED TO HER DEATH………………………………………………. |  | ECTOPIC |

| CALCULATE THE TOTAL NUMBER OF PREGNANCIES SHE HAS HAD, THAT IS THE SUM FOR 7.1 TO 7.8 CHECK THIS NUMBER WITH HER IN 7.9 AS FOLLOWS:…………………………. |  |  |  |
| --- | --- | --- | --- |

7.9 I would like to check with you the total number of pregnancies she had.

| From what you have told me, she had a total of [SUM] pregnancies, excluding the  pregnancy which led to her death. Is this correct?.......................................................... | 1. Yes | 2. No | CORRECT |
| --- | --- | --- | --- |

### IF THE ANSWER IS NO, REPEAT QUESTIONS 7.1 TO 7.8 UNTIL YOU HAVE AGREEMENT

| 7.10 In the past, did she ever have a caesarean section (NB: before the pregnancy which  led to her death?)………………………………………………………………………. | 1. Yes | 2. No | CS |
| --- | --- | --- | --- |

| 7.11 Before the pregnancy which led to her death, did she ever have a delivery where  the baby had to be pulled out with an instrument?......................................................... | 1. Yes | 2. No | VACUM |
| --- | --- | --- | --- |

| 7.12 DATE OF BIRTH OF LAST CHILD BEFORE THE PREGNANCY  WHICH LED TO HER DEATH [090909 = No child]……………… |  |  |  |  |  |  | DOBCHILD |
| --- | --- | --- | --- | --- | --- | --- | --- |

| 7.13 Where did she deliver her last child, before the pregnancy which led to her death?  [USE FACILITY KEY CODE; 98 = Home; 99 = NA, no child]……………………………………….. |  |  | WHEREDEL |
| --- | --- | --- | --- |

END OF ADULT FEMALE VA FORM. CHECK YOUR FORM AND THANK THE RESPONDENT

| **Certified correct on:** |  |  |  |  |  |  |  |  | **By:** |  |  | CCB |
| --- | --- | --- | --- | --- | --- | --- | --- | --- | --- | --- | --- | --- |
